# Sevoflurane ameliorates cerebral ischemia–reperfusion injury by modulating mitochondrial dynamics and attenuating apoptosis via Shh-YAP1 signaling pathway

**DOI:** 10.1186/s13041-026-01312-3

**Published:** 2026-05-21

**Authors:** Li Peng, Mengmeng Yang, Jiayuan Liu, Tianyue Yin, Jun Li, Shuaijie Sun, Hongrui Zhu, Sheng Wang

**Affiliations:** 1 Department of Anesthesiology, The First Affiliated Hospital of USTC, Division of Life Sciences and Medicine, University of Science and Technology of China, 16 Lujiang Road, Hefei City, 230001 Anhui People’s Republic of China; 2 Core Facility Center, The First Affiliated Hospital of USTC (Anhui Provincial Hospital), Hefei, 230001 Anhui People’s Republic of China; 3 Department of Anesthesiology, Wannan Medical College, Wuhu, 241002 People’s Republic of China

**Keywords:** Sevoflurane, Ischemic stroke, Sonic hedgehog, Mitochondrial dynamics, SUMOylation, Apoptosis

## Abstract

Ischemia–reperfusion injury (IRI) is a critical issue in the prevention and treatment of ischemic stroke. Inhalational anesthetics have been proven to have neuroprotective effects and sevoflurane is currently the most commonly used inhaled anesthetic in the clinic. However, the effects and underlying mechanism of sevoflurane on cerebral IRI have not been fully elucidated. In this study, oxygen–glucose deprivation model was established in primary mouse cortical neurons and HT22 cells, while the middle cerebral artery occlusion model was established in mice. Using laser speckle imaging, TTC staining, HE staining, Nissl staining, flow cytometry, calcium imaging and neurologic deficit scoring, we found that sevoflurane post-conditioning (SPC) significantly increased cerebral blood flow, reduced the infarct volume, alleviated neuronal pathological damage, promoted the survival of cortical neurons, reduced cell apoptosis, enhanced calcium responses and decreased the neurologic deficit scores after IRI. The results of RNA sequencing, Western blot, co-immunoprecipitation, TUNEL staining, immunofluorescence, transmission electron microscopy imaging, MPTP measurement, MMP detection, and ATP production measurement showed that the sonic hedgehog (Shh) signaling pathway crosstalk with Hippo-YAP signaling pathway, and the Shh-YAP1 pathway regulated mitochondrial dynamics and mitochondrial ultrastructure and function. In addition, SPC affected the phosphorylation and SUMOylation of dynamin-related protein 1 (Drp1), and there was an interaction between the phosphorylation and SUMOylation of Drp1. In conclusion, this study revealed that SPC might affect the phosphorylation and SUMOylation of Drp1 through the Shh-YAP1 signaling pathway, regulating mitochondrial dynamics, reducing cell apoptosis and ameliorating IRI. These findings offer new insights into the therapeutic strategies for ischemic stroke.

**Graphical abstract:**

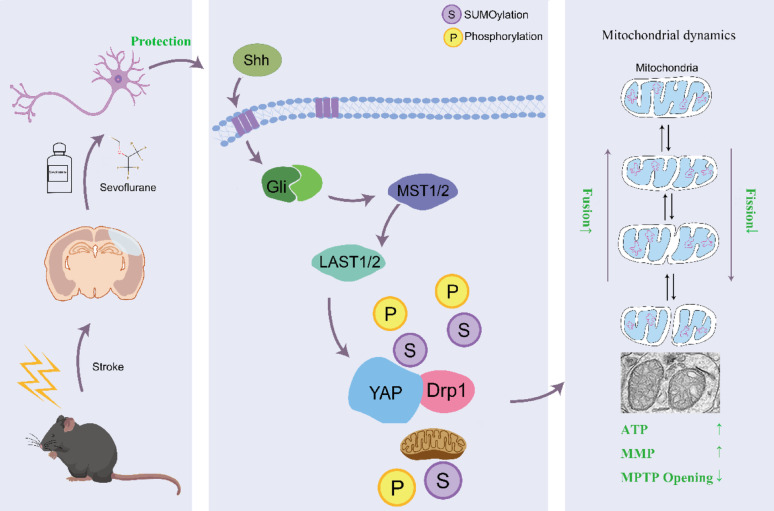

**Supplementary Information:**

The online version contains supplementary material available at 10.1186/s13041-026-01312-3.

## Introduction

The incidence of stroke is becoming younger, with ischemic stroke accounting for about 80% of all the stroke cases [1]. Currently, stroke has become the disease with the largest disability adjusted life year (DALY) among neurological diseases in the world. Early restoration of cerebral blood perfusion is key in treating ischemic stroke. The clinical interventions predominantly involve intravenous thrombolysis and mechanical thrombectomy to restore blood supply, but the reperfusion of blood supply will inevitably lead to secondary neuronal injury [2]. This phenomenon, wherein the restoration of blood flow exacerbates local tissue injury and functional impairment, is termed IRI. The pathophysiological process of cerebral IRI involves various mechanisms, including apoptosis, mitochondrial failure, Ca^2+^ overload, ROS accumulation and inflammatory responses. Currently, the mechanisms of cerebral IRI still require further research. Therefore, actively exploring effective interventions and their mechanisms for cerebral IRI is a significant clinical need to provide new treatment strategies for this condition.

Inhalational halogenated anesthetics such as isoflurane and sevoflurane are primarily used for general anesthesia, with sevoflurane being the most commonly used inhalational anesthetic in clinical practice today [3–5]. Our previous studies have also proved that isoflurane post-conditioning reduces apoptosis by regulating signaling pathways and provides significant protection against focal cerebral IRI [6, 7]. The neuroprotective effect of isoflurane is associated with the activation of the Shh-Gli1 signaling pathway in the hippocampal CA1 region and cortical neurons. The hedgehog signaling pathway is crucial for embryonic development, proliferation, apoptosis and angiogenesis [8]. Under effective stimulation, the secretion of Shh can activate the canonical hedgehog signaling pathway [9]. Shh binds to the receptor Patched via either autocrine or paracrine secretion, alleviating the inhibitory effect on the signal transducer protein Smoothened, allowing the transcriptional activator Gli1 to enter the cell nucleus and initiate the expression of target genes. These encourage us to position sevoflurane as a candidate for therapeutic testing in the context of cerebral IRI.

As is well known, the human brain comprises only 2% of adult body weight, yet it consumes 20% of the total energy demand of the body [10]. The brain lacks energy reserves and mitochondria are critical mediators of neuronal energy production [11]. The dynamic adaptation of mitochondria to changes in cellular energy demand through the continuous restructuring of mitochondrial network, and the ongoing processes of fusion and fission, is referred to as mitochondrial dynamics (mitochondrial fusion and fission) [12]. Cerebral IRI can disrupt the homeostasis of the intracellular environment and lead to neuronal death and neurological dysfunction by affecting the expression levels and post-translational modifications (PTMs) of proteins of mitochondrial dynamics-related proteins, causing mitochondrial fragmentation and dysfunction. Therefore, maintaining the balance of mitochondrial dynamics is an effective strategy for the prevention and treatment of cerebral IRI [13–15]. Drp1 is the core protein of mitochondrial division, and the translocation of Drp1 within mitochondria is a key step in inducing mitochondrial fission, which is primarily regulated by phosphorylation and SUMOylation. Complex interactions exist between different types of PTMs, collectively influencing the progression of diseases. However, the interaction between Drp1 phosphorylation and SUMOylation modifications during cerebral IRI, as well as the effects of SPC on the PTMs, has yet to be elucidated.

In order to explore the molecular mechanisms underlying the neuroprotective effects of SPC, we utilized the RNA sequencing technology and bioinformatics analysis, which indicated that SPC was related to the Shh signaling pathway, Hippo/YAP signaling pathway and apoptosis during cerebral IRI. The Hippo/YAP signaling pathway, belonging to the developmental biology signaling pathways along with the Shh signaling pathway [16], is also a crucial signaling pathway mediating cellular proliferation and apoptosis [17–21], with interactions observed between the two pathways [22]. In the carbon tetrachloride-induced liver fibrosis model, the Shh-YAP signaling pathway regulates hepatic ductular reactions during liver injury [23], while the Hedgehog-YAP signaling pathway controls hepatic stellate cell activation by regulating glutamine breakdown. Moreover [24, 25], Studies have shown that Hippo-YAP signaling pathway can affect mitochondrial dynamics by affecting the expression of Drp1. Therefore, we further hypothesized that the Shh-YAP-Drp1 axis may also be, at least partially, involved in the mechanisms underlying SPC against cerebral IRI.

In this study, we explored the role of the Shh-YAP1 signaling pathway in improving cerebral IRI after SPC treatment using a mouse middle cerebral artery occlusion/reperfusion model (MCAO/R) model and an oxygen–glucose deprivation/reoxygenation (OGD/R) model with primary cortical neurons and HT22 cells. We further investigated the potential molecular mechanisms by which SPC reduces apoptosis via Shh-YAP1 pathway by regulating mitochondrial dynamics and the phosphorylation and SUMOylation of Drp1. Here, we provided evidence for the treatment of experimental cerebral IRI with SPC and new theoretical foundations and technical support for the prevention and treatment of ischemic stroke.

## Material and methods

### Animals and ethics

Adult male C57BL/6J (RRID: IMSR_JAX:000664) mice were purchased from Beijing Vital River Laboratory Animal Technology Co Ltd (Beijing, China, license number: SCXK 2021-0006) wild-type mice, 8–10 weeks old, weighing 23 ± 2g, were kept in a constant temperature, quiet and clean environment. Animals can eat and drink freely, automatically control the diurnal cycle (12/12h), and the room temperature is (22 ± 2) ℃. Mice were fasted for 2 h before surgery and acclimated to the experimental environment in advance. All animal procedures in this study were approved by the Ethics Committee of the First Hospital of USTC (Anhui Provincial Hospital) (No.2022-N-124).

### Establishment of MCAO/R model

Mice were anesthetized by intraperitoneal injection of 2,2,2-tribromoethanol (Cat#: HY-B1372, 250 mg/kg, MedChemExpress, USA) and then fixed in the supine position. A midline incision was made in the neck, and the subcutaneous tissue was dissected to expose and separate the right common carotid artery, external carotid artery, and internal carotid artery. A small notch was made in the external carotid artery, and the filament (Cat#: MSMC21B120PK50, RWD Life Science, China) was passed from the external carotid artery into the internal carotid artery to the middle cerebral artery. When resistance was felt at a depth of approximately 0.8 cm from the bifurcation of the common carotid artery, the thread advancement was stopped. Subsequently, the incision of the external carotid artery was ligated, the skin was sutured, and the mice were placed in a 37 °C constant temperature chamber. After 1 h of ischemia, the thread was withdrawn to perform cerebral tissue reperfusion. This study excluded mice that died, suffered surgical failures, or experienced subarachnoid hemorrhage.

### Cell culture and establishment of OGD/R model

Primary cortical neurons were derived from the cerebral cortex of newborn mice within 1 d. After disinfection of newborn mice, the whole brain was stripped under sterile conditions and the meninges, hippocampus and cerebellum were removed. Rinsing with DMEM/F12 medium (Cat#: 11320033, Gibco, USA), cut it into pieces and add 0.125% trypsin digestion solution (Cat#: 25200056, Gibco, USA). Digestion was terminated with DMEM/F12 medium containing 10% fetal bovine serum (FBS, Cat#: 16000044, Gibco, USA) after 15 min in an incubator at 37 ℃. Then the cells were filtered with a 200-mesh screen, the filtrate was centrifuged and the supernatant was removed. The cells were seeded in 6-well plates with DMEM/F12 medium containing 10% fetal bovine serum was added. The medium was replaced with Neurobasal medium after 6 h and the medium was changed every 3 d for a total of 7 d.

HT22 cells (Cat#: 3101MOUGNM47, C5, RRID: CVCL_0321) were purchased from Cell Resource Center, Peking Union Medical College. Cells were cultured in high glucose DMEM containing 10% FBS, and 1% penicillin–streptomycin (Cat#: 15140122, Gibco, USA) and changed the medium every two days.

In vitro simulation of cerebral IRI requires the establishment of OGD/R models. In short, after washing the cells with phosphate-buffered saline (PBS, Cat#: G4202-500ML, Servicebio, China) twice, the medium was replaced by glucose-free DMEM (Cat#: 11966025, Gibco, USA) without FBS. Then, the cells were exposed to hypoxia (1% O_2_, 5% CO_2_, and 94% N_2_) for 1 h followed by reoxygenation (95% air, 5% CO_2_) for 24 h.

### RNA interference and plasmid transfection

The plasmids WT-Drp1, dephosphorylation‐mimic of p-Drp1S616 mutants (Drp1-S616A) and non-SUMOylated Drp1-8KR mutant (8KR, K532R, K535R, K558R, K568R, K594R, K597R, K606R, K608R) were constructed by GeneChem (Shanghai, China). The HT22 cells were transfected with plasmids or shRNA using Lipofectamine 2000 Reagent (Cat#: 11668-027, Invitrogen, USA) according to the manufacturer’s instructions.

### SPC in vivo and in vitro

The protocol of SPC retreatment was similar to that of previous in vivo studies [6, 8]. Sevoflurane (Cat#: H20070172, Hearem, China) was delivered with the vehicle air (30% O_2_ and 70% air) using an agent-specific vaporizer (Cat#: 891585-01, Datex Ohmeda, USA). Immediately at the beginning of reperfusion, mice were placed into the inhalation anesthesia device (patent number: ZL201520074763.0) for 1 h after the sevoflurane concentration reached a steady level of 2.5%. Concentrations of sevoflurane was measured continuously using the anesthetic gas monitor (Cat#: 8001595, Datex Ohmeda, USA) [26]. For in vitro experiment, SPC was carried out after OGD treatment, the cells were placed in a chamber with 2.5% sevoflurane in the carrier gas of 5% CO_2_, 95% air for 1 h at 37 °C. The concentration of sevoflurane in the chamber was determined by anesthetic gas analyzer.

### Stereotaxic surgeries and microinjections

The mice were anesthetized and immobilized in a stereotaxic head frame (Cat#: 68801, RWD Life Science, China) while maintaining body temperature using a heating pad. Ophthalmic ointment was applied to the eyes to prevent drying. Before scalp incision, lidocaine was injected for local anesthesia. A Micro Sample Syringes was used to deliver the adeno-associated viruses (AAV) or control vehicle to CA1 and inject the virus. CA1 coordinates were − 2 A/P, − 1.5 M/L at depths − 1.5 D/V with respect to bregma. The needle was held in place for 10 min before slowly removing from the brain using a stereotactic injection apparatus (Cat#: 788130, RWD Life Science, China).

### RNA interference and calcium imaging

For RNA interference [27], mice were randomly infected with AAV-Control-EGFP or AAV-YAP1-shRNA-EGFP (Virus titer: 1.5 × 10^12^ vg/ml, Hanbio Co. Ltd, Shanghai, China). For calcium imaging, rAAV-CaMKIIα-GCaMP6f (Cat#: BC-0083) were packaged and supplied by Brain Case Biotechnology (Wuhan, China) at titers 2.67 × 10^12^ vg/ml [28]. The stereotaxic surgeries and microinjection injection procedures of the two viruses were similar. For the mice used for calcium imaging, the fiber optic cannulas were affixed to the skull using light-cured dental composite and dental acrylic. Three weeks after the AAV injection, Mice were subjected to MCAO and calcium signaling experiments were conducted using a fiber-optic recording system (Cat#: FPS-410/470/561, Inper, China).

### RNA sequencing

Total RNA was extracted and the purity, quantification and integrity of RNA were assessed. The raw data obtained by sequencing were filtered and the filtered clean reads were compared to the reference sequence. Based on the comparison results, new transcript prediction, SNP and Indel detection were analyzed. In addition, quantitative analysis of known genes and new genes was conducted, differential expression analysis was conducted according to the expression levels of genes in different sample groups, gene ontology (GO) and kyoto encyclopedia of genes and genomes (KEGG) enrichment analysis, cluster analysis, protein interaction network, transcription factor coding ability prediction and other further mining and analysis were conducted for the selected differentially expressed genes (DEGs).

### Laser speckle imaging

Mice were anesthetized and secured in the stereotaxic frame positioned under a laser speckle contrast imaging (LSCI) instruments (Cat#: RFLSI Ⅲ, RWD Life Science, China) through the intact skull to image the cerebral blood flow (CBF) changes before and after MCAO.

### Behavioral testing

In order to determine the neurological function injury of mice [29], Bederson and modified Longa scores were performed 24 h after reperfusion by observers who did not know the experimental conditions. Higher scores in mice indicate more severe neurological deficits [30]. The Y-Maze novel arm exploration experiment tested learning and memory by taking advantage of the fact that mice prefer to spend time in new areas. The three arms of the Y maze are named start arm, novel arm and other arm respectively. The mice need to be adapted and trained first. After the new arm was separated by a partition, the mice were put into the other two arms to move freely for 10 min. The baffle was removed for testing after 1 h, and the mice were randomly placed in the arm and moved freely for 5 min. Data statistics: The frequency with which the mice entered each arm and the time spent exploring each arm. The testing apparatus was cleaned with 75% ethanol between mice. The modified neurological severity scoring (mNSS) test is a composite of motor, sensory, reflex, and balance tests. Neurological function was graded on a scale of 0–18, with higher scores indicating more severe dysfunction. For the rotarod test, the rats in each group were trained for three consecutive days before stroke. Three measurements at predetermined intervals were taken with an automated rotarod meter (Cat#: XR-6C, Xinxin Life Science, China), and the mean latency time to fall was calculated. The adhesive removal test is a sensitive method to assess sensorimotor deficits in mice. To assess the somatosensory dysfunction following cerebral ischemia, we performed the adhesive removal (sticky-tape) test. Two 2 × 3 mm sticky tapes were applied to the forepaws and tactile responses were measured by recording the time to remove the adhesive tape. Pre-training for 7 days was conducted before MCAO.

### Measurement of infarct volume

Mice brains were removed and slices were taken at 1 mm intervals rapidly after being deeply anesthetized. The sections were immersed in 2% 2,3,5-triphenyl tetrazolium chloride (TTC, Cat#: 2273GR025, Biofroxx, Germany) staining solution at 37 °C for 20 min [7]. The infarct volume was measured and analyzed as previously described.

### Histopathological assessment

Mice brains were removed after transcardially perfused with PBS followed by 4% Paraformaldehyde (PFA, Cat#: YB36315ES60, Sigma, USA) and fixed in 4% PFA for 24 h before embedded in paraffin. Paraffin specimens (5 μm), stained with nissl staining (toluidine blue method) and hematoxylin and eosin (H&E) staining were analyzed blindly.

### Immunofluorescence and TUNEL staining

The samples (cells or perfused mice brains) were fixed in 4% PFA at room temperature, followed by permeabilization with 0.3% Triton X-100 in PBS for 15 min. After blocking with 5% bovine serum albumin, Terminal Deoxynucleotidyl Transferase-Mediated dUTP Nick End Labeling (TUNEL) assay was performed using an In Situ Cell Death Detection Kit (Cat#: 11684817910, Roche, Germany) according to the manufacturer's instructions. The samples were subsequently incubated with primary antibodies in a humidified chamber at 4 °C overnight, washed in PBS for three times, and incubated with secondary antibodies for 1 h at room temperature. Next, the cellular nuclei were stained with DAPI solution for 5 min in the dark. Finally, the images were captured using a confocal laser scanning microscope (Cat#: LSM800, Zeiss, Germany).

### Cell apoptosis assay

After OGD/R treatment, the HT22 cells were collected and stained by an Annexin V-FITC/PI Apoptosis Detection Kit (Cat#: C1062M, Beyotime, China). Apoptosis induced by OGD/R was measured by a BD LSRFortessa flow cytometer (Cat#: FACSFortessa, Becton Dickinson, USA).

### Quantitative PCR

Priming sequences of YAP1 and GAPDH were designed, and RNeasy Mini Kit (Cat#: 74104, Qiagen, Germany) was used to extract RNA from brain tissues of mice according to the manufacturer's instructions. In this experiment, the primer sequences for YAP1 were: forward 5′-gccatgctttcgcaactgaa-3′ and reverse 5′-actctgtgatcctctggttc-3′. The primer sequences for the reference GAPDH were: forward 5′-AGGTCGGTGTGAACGGATTTG-3′ and reverse 5′-GGGGTCGTTGATGGCAACA-3′.Then quantitative PCR was performed using HiScript Q RT SuperMix (Cat#: R223-01, Vazyme, China) and AceQ qPCR SYBR Green Master Mix (Cat#: Q111-02, Vazyme, China) on a machine (Cat#: LightCycler 480II, Roche, Germany), and the relative expression level was calculated using 2^−△△CT^ method.

### Western blot

Proteins were extracted from mice brain tissue or cells, and the concentration was detected using the BCA protein assay kit (Cat#: P0012, Beyotime, China). The proteins were separated by sodium dodecyl sulfate–polyacrylamide gel electrophoresis (SDS-PAGE) and transferred to polyvinylidene fluoride (PVDF) membranes. After 2 h with 5% skim milk powder at room temperature, the PVDF membranes were combined with primary antibodies (1:1000) overnight at 4 °C. The primary antibodies used in this study were as follows: Shh (Cat#: sc-365112, RRID: AB_10709580), Gli1 (Cat#: sc-515751, RRID: AB_2934097), YAP1 (Cat#: sc-101199, RRID: AB_1131430), Mfn1 (Cat#: sc-166644, RRID: AB_2142616), Mfn2 (Cat#: sc-100560, RRID: AB_2235195), OPA1 (sc-393296, RRID: AB_3101815), Fi8 (Cat#: sc-376447, RRID: AB_11149382), t-Drp1 (Cat#: sc-271583, RRID: AB_10659110), and Bcl-2 (Cat#: sc-7382, RRID: AB_626736) were purchased from Santa Cruz (Dallas, USA); MST1 (Cat#: 3682, RRID: AB_2144632), LATS1 (Cat#: 9153, RRID: AB_2296754), p-Drp1^S616^ (Cat#: 3455, RRID: AB_2085352), p-Drp1^S637^ (Cat#: 4867, RRID: AB_10622027) and Cle.caspase-3 (Cat#: 9664, RRID: AB_2070042) were purchased from Cell Signaling Technology (Boston, USA); SUMO1 (Cat#: AF0280 RRID: AB_2833449), Pro.Caspase-3 (Cat#: AF6311, RRID: AB_2835170) and Caspase-9 (Cat#: AF6348, RRID: AB_2835042) was purchased from Affinity Biosciences (Beijing, USA); SUMO2/3 (Cat#: A5199, RRID: AB_3731315), UBC9 (Cat#: F14C23, RRID: AB_3731317), and Bax (Cat#: F0037, RRID:AB_3698184) were purchased from Selleck Chemicals (Houston, USA); GAPDH (Cat#: 60004-1-Ig, RRID: AB_2107436) and β-actin (Cat#: 20536-1-AP, RRID: AB_10700003) were purchased from Proteintech (Chicago, USA). After washing with TBST for three times, the membrane was incubated with goat anti-rabbit IgG-HRP secondary antibody (1:10000, Cat#: C31460100, ThermoFisher, USA) or goat anti-mouse IgG-HRP secondary antibody (1:10000, Cat#: C31430100, ThermoFisher, USA) at room temperature for 2 h, and then treated with ECL reagent (Cat#: 34579, ThermoFisher, USA) to detect protein expression levels. Using Image J software, the band gray values were normalized to GAPDH or β-actin levels.

### Co-immunoprecipitation (Co-IP)

The cells or right cerebral cortices located in ischemic penumbra were added to the lysis buffer and the supernatant was obtained by centrifugation. 200 μl of lysate was used for Western blot analysis, and the remaining lysate was added with 1 μg IgG and YAP1 or Drp1 primary antibody and incubated for 3 h. Then 50 μl protein A/G agarose beads were added and incubated overnight at 4 °C. After immunoprecipitation reaction, agarose beads were centrifuged to the bottom of the tube, and finally the SDS sample loading buffer was boiled for Western blot analysis.

### Transmission electron microscope (TEM) imaging

The right cerebral infarction tissues were fixed with 2% PFA and 3% glutaraldehyde in PBS for 2 h, and then overnight at 4 °C. The sections were embedded with epon, stained with 1.5% potassium ferricyanide and 2% uranate lactate, and the changes of mitochondrial structures observed by TEM (Cat#: Tecnai T12 120kv, ThermoFisher, USA).

### Adenosine triphosphate (ATP) detection

The cells were lysed by adding 200 μl lysis buffer to each well of the 6-well plates. After lysis, the samples were centrifuged at 12,000 g for 5 min at 4 °C to collect the supernatant. The intracellular concentration of ATP was measured by an ATP Assay Kit (Cat#: S0026, Beyotime, China). The absorbance is measured with an enzyme-labeled instrument, and the result was expressed as the ratio of the test value to the control value.

### Measurement of mitochondrial membrane potential (MMP)

We used a JC-1 kit (Cat#: BL711A, Biosharp, China) to measure the MMP. After OGD/R injury in vitro, the collected cells were washed with PBS twice and then incubated with JC-1 staining buffer in the cell incubator for 20 min. After incubation, the solution was replaced with fresh medium and the fluorescence of the sample was measured by fluorescence microscope (Cat#: Axio Imager M2, Zeiss, Germany) and quantified by Image J software. The complexes with red fluorescent were formed in cells with normal mitochondria. In mitochondria-damaged cells, JC-1 maintained a monomeric form and showed green fluorescence. Mitochondrial potential is expressed as relative aggregate-to-monomer (red/green) fluorescence intensity ratio.

### Measurement of mitochondrial permeability transition pore (MPTP) opening

We employed the Calcein AM and CoCl_2_ assay kit (Cat#: BL928A, Biosharp, China) to detect the opening of MPTP. After OGD/R injury in vitro, the collected cells were washed with PBS twice and then incubated with JC-1 staining buffer in the cell incubator for 20 min. The detection of MPTP was similar to that of MMP. In short, Calcein-AM staining solution, fluorescence quenching working solution or Ionomycin control were added to the cells and incubated in the cell incubator for 30 min. After incubation, the solution was replaced with fresh medium and incubated for another 30 min. Finally, the fluorescence of the sample was measured by fluorescence microscope and analyzed by Image J software.

### Proximity ligation assay (PLA)

The cells were fixed in 4% PFA at room temperature, followed by permeabilization with 0.3% Triton X-100 in PBS for 15 min. After blocking with Duolink® Blocking Solution, PLA was performed using an In Situ Red Starter Kit (Cat#: DUO92008, Sigma-Aldrich, USA) according to the manufacturer's instructions. The samples were subsequently incubated with anti-YAP1 and anti-TOM20 (Cat#: A19403, RRID: AB_2862646) in a humidified chamber at 4 °C overnight, washed in wash buffer for three times, and incubated with PLA Mouse and Rabbit probes (Cat#: DUO92002 and DUO92004, Sigma-Aldrich, USA) for 1 h at 37 °C. Subsequently, cells were sequentially subjected to ligation and amplification according to the instructions. Finally, the cellular nuclei were stained with DAPI solution for 5 min in the dark, and fluorescence images were captured using a fluorescence microscope.

### Statistical analysis

Experiments were repeated at least 3 times for each test, and the data are presented as the means ± SD. Normality was assessed using the Shapiro–Wilk test. For data with normal distribution, Student's t test was used for comparison between two groups, and one-way analysis of variance (ANOVA) followed by Bonferroni's multiple comparison test for comparison among three or more groups, using GraphPad Prism 8.0 or SPSS version 17.0. The investigators analyzing the data were blinded in terms of the experimental conditions.

## Results

### SPC increases cerebral blood flow, reduces infarct volume and improves histopathological injury

**Fig. 1:**
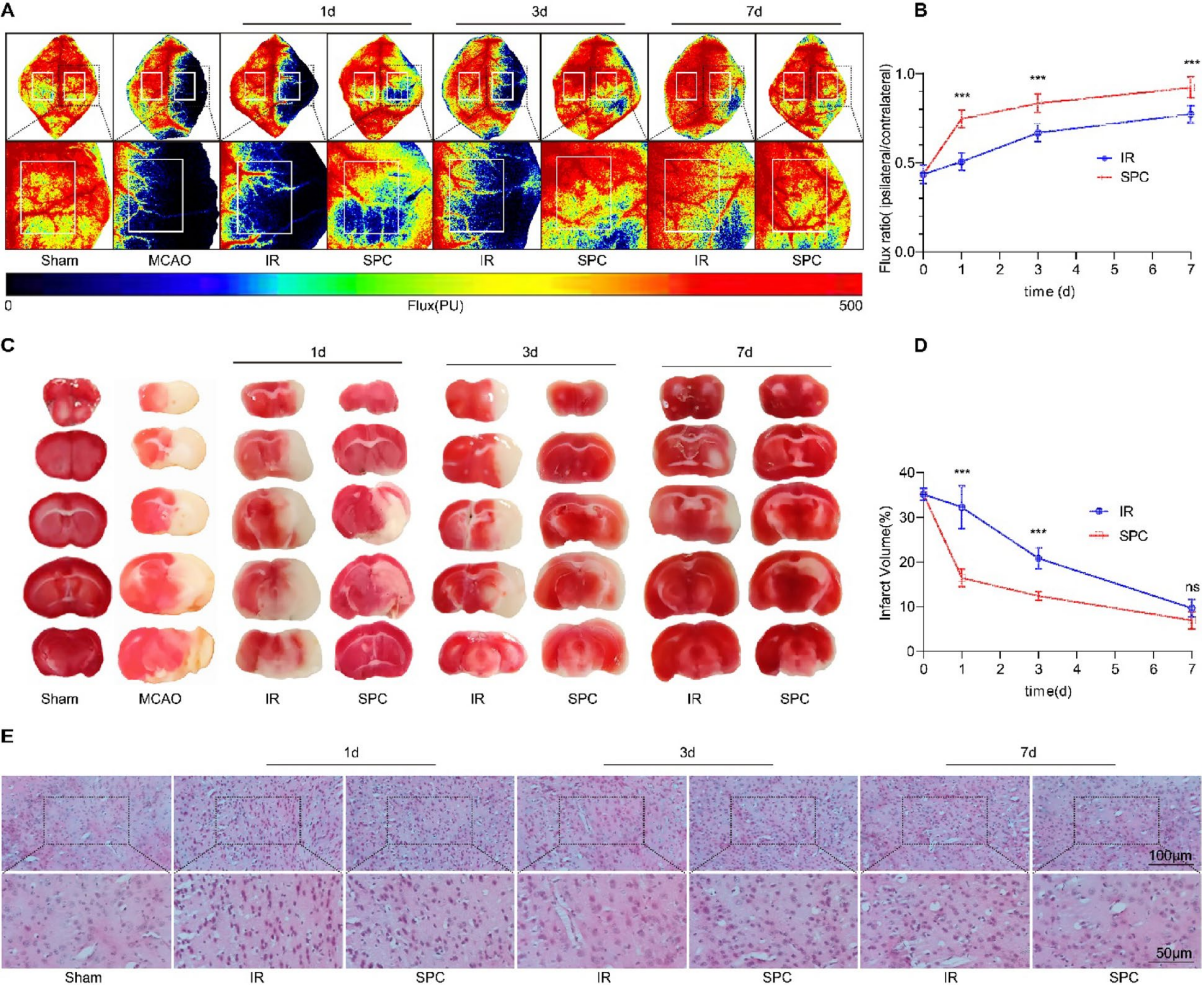
SPC increased cerebral blood flow, reduced infarct volume and improved histopathological injury in the infarcted cortex. **A** Representative LSCI raw data with defined ipsilateral and contralateral regions of interest (ROIs). **B** Flux ratios at 1 d, 3 d, and 7 d after MCAO. **C**–**D** TTC staining and analysis of infarct volume at 1 d, 3 d, and 7 d after IRI. **E** Typical HE staining images at 1 d, 3 d, and 7 d after IRI, with scales of 100 µm and 50 µm. Data are presented as the mean ± SD. Non-significant (ns), *P < 0.05, **P < 0.01, *** *P* < 0.001, compared to the IR group

After the MCAO model is successfully established, we observed the mean flux measurements determined using LSCI were reduced by approximately 50% in the ROI encompassing the MCA territory of the ipsilateral hemisphere compared with an equivalent ROI in the contralateral hemisphere (n = 6 mice per group, two-tailed *t* test, t = 25.98, *p* < 0.0001, Fig. 1A, B). Flux ratios were significantly increased in the SPC group compared to that in the IR group at 1 d (n = 6 mice per group, two-tailed *t* test, t = 10.69, *p* < 0.0001), 3 d (n = 6 mice per group, two-tailed *t* test, t = 7.167, *p* < 0.0001), and 7 d (n = 6 mice per group, two-tailed *t* test, t = 5.080, *p* = 0.0005) after IRI (Fig. 1B). The results of TTC staining showed that the infarct volume was significantly reduced in the SPC group compared to that in the IR group at 1 d (n = 6 mice per group, two-tailed *t *test, t = 8.775, *p* < 0.0001) and 3 d (n = 6 mice per group, two-tailed *t* test, t = 5.080, *p* = 0.0003) after IRI (Fig. 1C, D). HE staining showed that histopathology of the cortex was normal in the Sham group. In the IR group, cells were disordered and necrotic cells was observed. Significant histopathological improvement was observed in the SPC group compared to that in the IR group at 1 d and 3 d after IRI (Fig. 1E and Fig. S2 A). We found that SPC improved significantly within 3 d after cerebral IRI, especially on the first day. Therefore, 24 h was chosen as the observation period for experiments to study the role of SPC after cerebral IRI.

### SPC inhibits apoptosis, promotes neuronal survival and improves the neurological function in the early phase of cerebral IRI

**Fig. 2:**
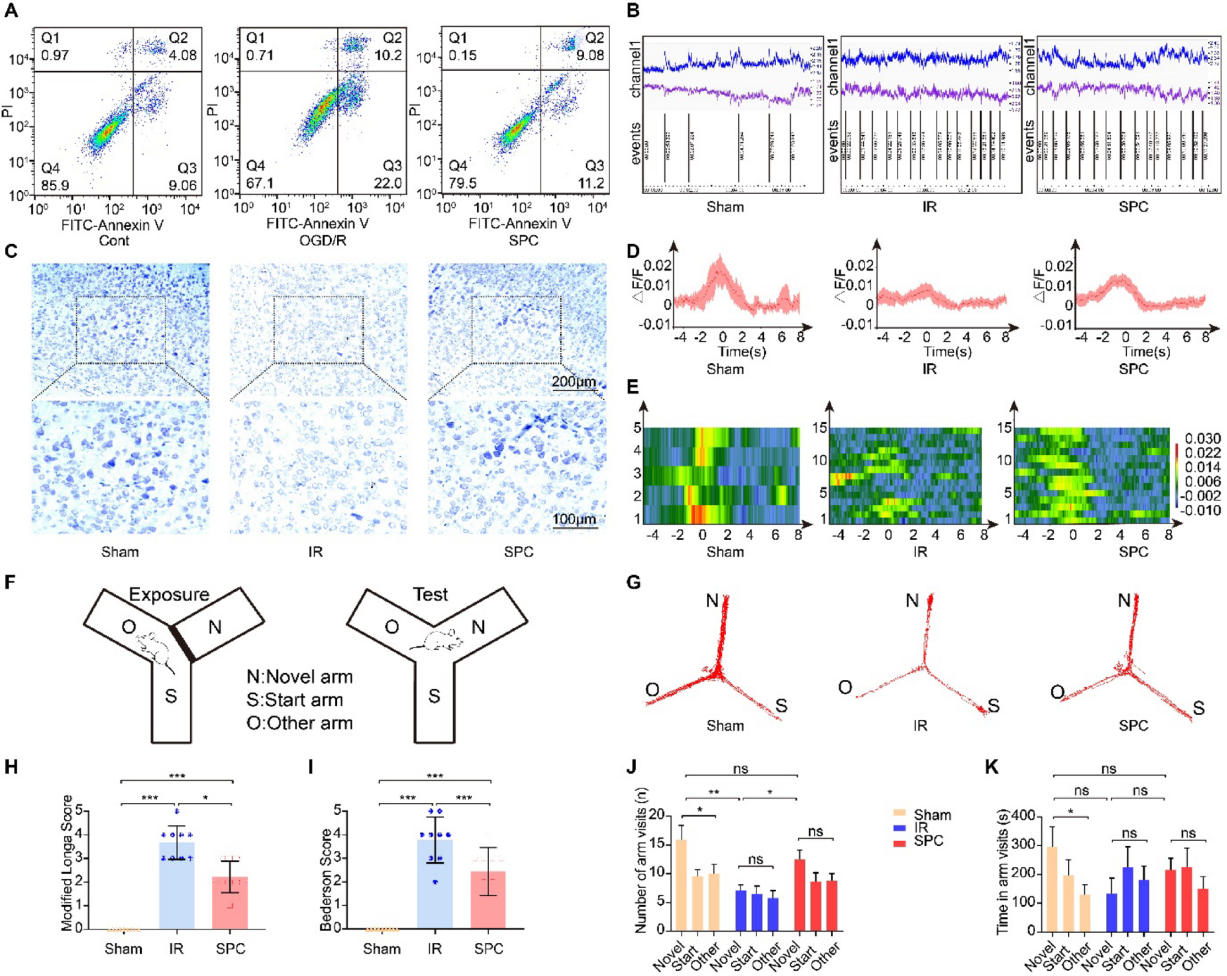
SPC inhibits apoptosis, promotes neuronal survival and improves the neurological function. **A** The apoptosis of HT22 cells was detected by flow cytometry. **B** Imaging of calcium activity in mice subjected to odor exposure. **C** Histological images of Nissl staining evaluated the neuronal survival in the ipsilateral cortex, with scales of 200 µm and 100 µm. **D** The mean calcium responses of hippocampal CA1 pyramidal neurons. The formula for ΔF/F0 is (F-F0)/F0, the baseline (F0). **E** Heat maps of the mean calcium responses. (F) Simplified experimental schematic diagram of Y-Maze. **G** Illustration of motion trajectory among Sham, IR, SPC groups at 1 d after stroke. (H) Neurological function scores with the modified Longa score. **I** Neurological function scores with the Bederson score. **J** The number of novel arm visits the mice made during the Y-Maze experiment. **K** Time taken for mice to visit novel arm during the Y-Maze experiment. Data are presented as the mean ± SD. Non-significant (ns), *P < 0.05, **P < 0.01, *** *P* < 0.001

The results of cell apoptosis experiment showed that the apoptosis of HT22 cells was significantly increased after OGD/R, especially the early apoptotic cells in Q3 region, while SPC reduced the apoptosis, indicating that SPC inhibited apoptosis mainly by affecting the number of early apoptotic cells, but had little effect on late apoptotic and necrotic cells (Fig. 2A). Next, ^31^we monitored the activity of hippocampal CA1 pyramidal neurons in mice expressing the calcium indicator GCaMP6f, using calcium imaging. The neural responses decreased significantly after IRI, while the neural responses increased after SPC (Fig. 2B, D, E). The results of Nissl staining were similar. Nissl staining showed that the Nissl bodies staining was lightly stained in the IR group, while the SPC group had deeper Nissl bodies staining and significantly increased the number of survival cells compared with the IR group (Fig. 2C). These improvements were further supported by the data analysis of the modified Longa score and Bederson score showed that SPC improved the neurological function and reduced the neurologic deficit scores in mice (n = 9 mice per group, two-tailed *t* test, t = 2.848, *p* = 0.0116, Fig. 2H and n = 9 mice per group, two-tailed *t* test, t = 4.459, *p* = 0.0004, Fig. 2I). In addition, SPC improved the spatial recognition memory ability evaluated by Y-Maze test in MCAO mice (Fig. 2F, G), showing significant increased number of novel arm visits compared with IR group (n = 9 mice per group, two-tailed *t* test, t = 2.852, *p* = 0.0128, Fig. 2J). However, there was no statistical difference of time in novel arm visits, although there was an increase trend SPC group (n = 9 mice per group, two-tailed *t* test, 133.1290 ± 152.3214 in the IR group, 216.4456 ± 110.9357 in the SPC group, *p* = 0.2316, Fig. 2K).

### SPC functions in cerebral IRI involved in the Shh signaling pathway and apoptosis

**Fig. 3:**
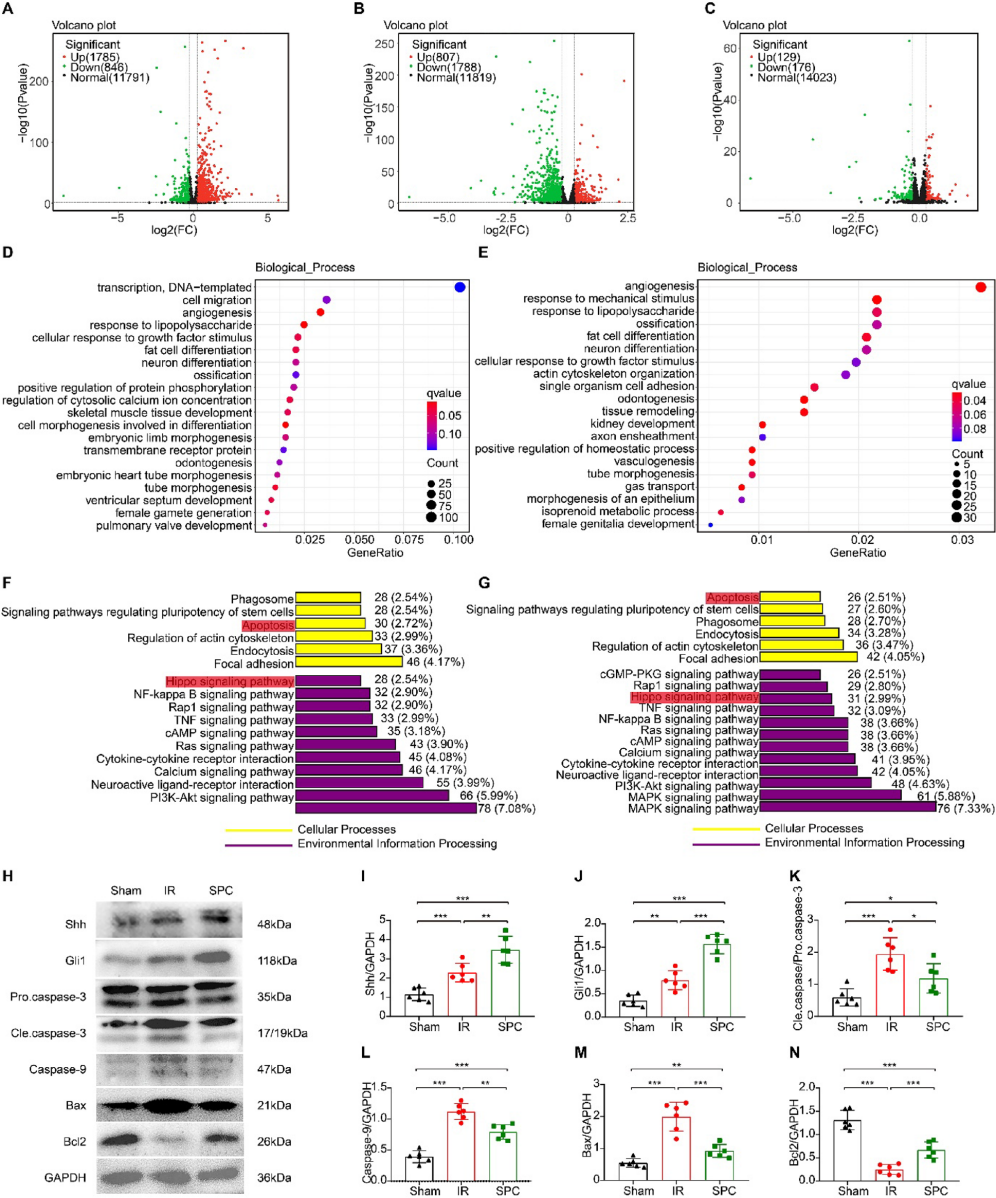
Joint analysis of signaling pathway expression and apoptosis-related protein changes based on data from RNA sequencing from mice brain tissues. **A** Volcano map of differentially expressed genes (DEGs) between Sham and IR groups. The green dots represent down-regulated DEGs, the red dots represent up-regulated DEGs, and the black dots represent the genes with insignificant difference. **B** Volcano map of DEGs between IR and SPC groups. **C** Volcano map of DEGs between Sham and SPC groups. **D** Gene Ontology(GO) enrichment analysis of DEGs between Sham and IR groups. **E** GO enrichment analysis of DEGs between IR and SPC groups. **F** The KEGG pathway types classification of DEGs between Sham and IR groups. **G** The KEGG pathway types classification of DEGs between IR and SPC groups. (H-N) Protein expression levels and analysis of Shh signaling pathway and apoptosis-related proteins in ischemic penumbra. Data are presented as the mean ± SD. *P < 0.05, **P < 0.01, ***P < 0.001

RNA sequencing technology followed by further bioinformatics analysis was performed to explore the mechanism of SPC protecting against cerebral IRI. We systematically studied transcriptomics and found that a large amount of DEGs was upregulated in the IR group compared with the Sham group (Up:1785, Down:846), while the difference in the number of DEGs in the SPC group was small compared with the Sham group (Up:129, Down:176), suggesting that SPC may play a protective role by normalizing the DEGs after IRI (Fig. 3A–C). We further used GO analysis of DEGs to characterize their respective biological functions. The highest correlation of biology-process-related genes were annotated with GO terms “angiogenesis" between the Sham and IR groups and between the IR and SPC groups (Fig. 3D, E). By searching the DEGs data, we found that Shh is a representative gene of angiogenesis in biological-process. The KEGG pathway type classification between Sham group and IR group, IR group and SPC group showed that DEGs were related to apoptosis and Hippo signaling pathway (Fig. 3F, G). Western blot analysis further confirmed that DEGs were associated with Shh signaling pathway and apoptosis-related proteins after cerebral IRI. Compared with Sham group, the expression levels of Shh and Gli1 in IR group (n = 6 mice per group, two-tailed *t* test, t = 4.752, *p* = 0.0008, Fig. 3I and n = 6 mice per group, two-tailed *t* test, t = 4.533, *p* = 0.0011, Fig. 3J) and SPC group (n = 6 mice per group, two-tailed *t* test, t = 7.334, *p* < 0.0001, Fig. 3I n = 6 mice per group, two-tailed *t* test, t = 12.390, *p* < 0.0001, Fig. 3J) were increased, indicating that the Shh signaling pathway was activated after IRI (Fig. 3H–J). At the same time, IRI increased the expression of pro-apoptotic proteins (n = 6 mice per group, two-tailed *t* test, t = 5.758, *p* = 0.0002, Fig. 3K n = 6 mice per group, two-tailed *t* test, t = 11.070, *p* < 0.0001, Fig. 3L n = 6 mice per group, two-tailed *t* test, t = 7.528, *p* < 0.0001, Fig. 3M) and decreased the expression of anti-apoptotic protein Bcl-2 (n = 6 mice per group, two-tailed *t* test, t = 11.090, *p* < 0.0001, Fig. 3N), and this effect was reversed by SPC (Fig. 3H, K–N). In fact, the pro-angiogenic and anti-apoptosis effects of Shh signaling pathway has been confirmed in many studies, and our previous studies have also shown that isoflurane post-conditioning further activates Shh signaling pathway after cerebral IRI and alleviates IRI by promoting VEGF expression and promoting angiogenesis.

### SPC reduces apoptosis and alleviates cerebral IRI through the Hippo-YAP signaling pathway

**Fig. 4:**
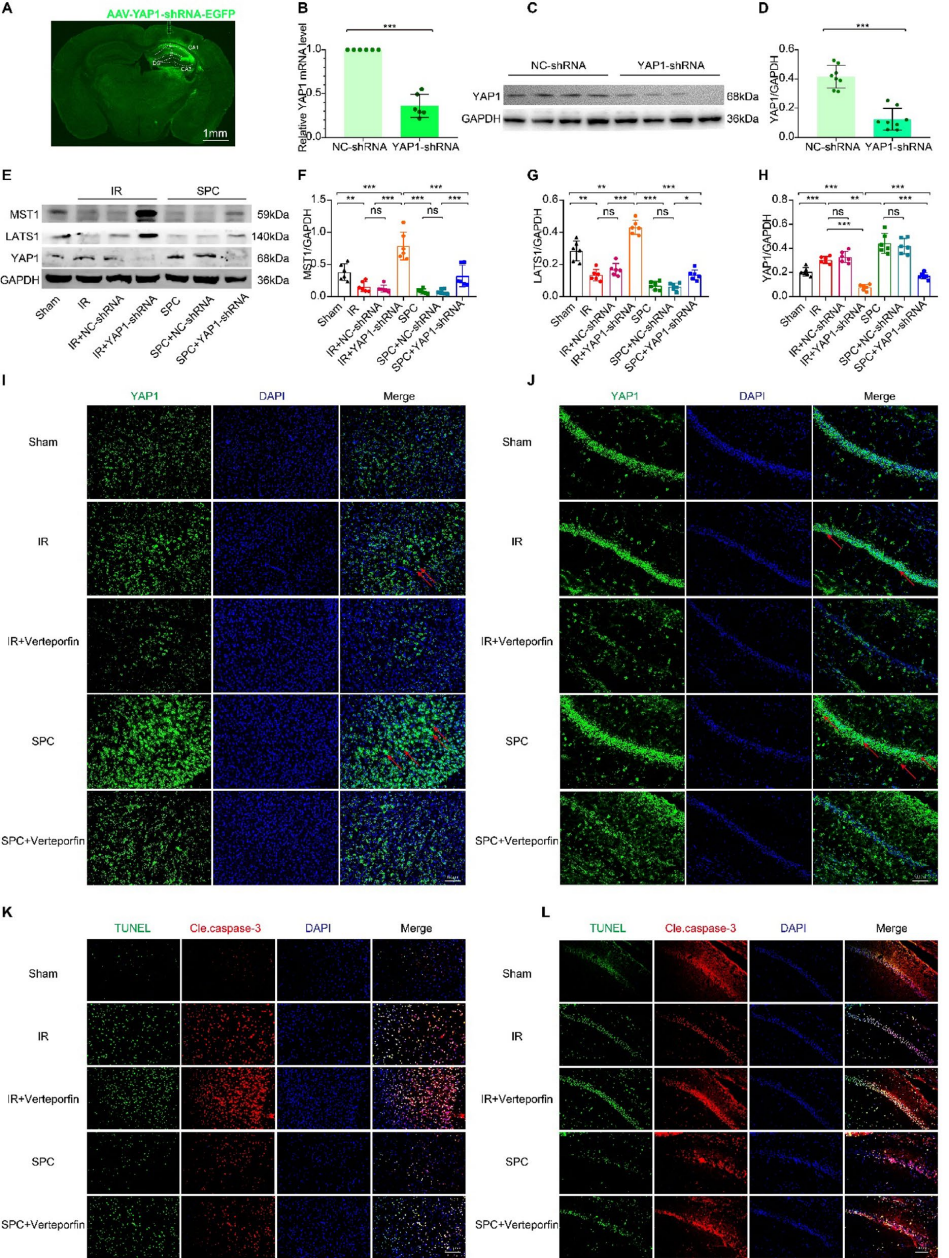
SPC reduces apoptosis and alleviates cerebral IRI through the Hippo-YAP signaling pathway in the ischemic penumbra and hippocampal CA1 region. **A** Hippocampus of mice infected with AAV-YAP1-shRNA-EGFP 3 weeks later. **B** The relative YAP1 mRNA level in hippocampus. **C–D** The expression level of YAP1 protein in hippocampus. **E**–**H** Protein expression levels and analysis of Hippo-YAP signaling pathway. **I** Immunofluorescence of YAP1 in the ischemic penumbra. Red arrows indicate the position of YAP1 with nuclear translocation. **J** Hippocampal CA1 region immunofluorescence of YAP1. **K**–**L** Representative images of TUNEL and Cle.caspase-3 double immunofluorescence in ischemic penumbra and hippocampal CA1 region. NC-shRNA was used as negative control. Data are presented as the mean ± SD. *P < 0.05, **P < 0.01, *** *P* < 0.001

As the results of RNA sequencing suggest that DEGs were related to apoptosis and Hippo-YAP signaling pathway, we intended to investigate whether Hippo-YAP signaling pathway is involved in the protective effect of SPC in IRI and whether this protective effect is related to apoptosis. First, we injected 1.5 μl AAV-YAP1-shRNA-EGFP into the hippocampus of mice to knockdown YAP1 (Fig. 4A), the prime transcriptional effector of Hippo-YAP signaling pathway, and tested the knockdown effect by real-time PCR and Western blot, confirming that the knockdown of YAP1 was significant (n = 6 mice per group, two-tailed *t* test, t = 11.950, *p* < 0.0001, Fig. 4. B and n = 6 mice per group, two-tailed *t* test, t = 7.672, *p* < 0.0001, Fig. 4C, D). Next, we detected the proteins expression levels of MST1, LATS1 and YAP1, the main components of Hippo-YAP signaling pathway. Under normal circumstances, the expression level of YAP1 was relatively low, but it increased and transitioned into an activated state after IRI (n = 6 mice per group, two-tailed *t* test, t = 5.353, *p* = 0.0003, Fig. 4E, H). SPC further enhanced the expression level of YAP1 (n = 6 mice per group, two-tailed *t* test, t = 3.896, *p* = 0.003, Fig. 4E, H), while knocking down YAP1 significantly reduced its expression level (n = 6 mice per group, two-tailed *t* test, t = 7.714, *p* < 0.0001, Fig. 4E, H). The expression trends of MST1 and LATS1 were opposite to that of YAP1 (Fig. 4E–G) and NC-shRNA had no effect on the protein expression levels of MST1 (IR group vs. IR + NC-shRNA group, n = 6 mice per group, two-tailed *t* test, t = 0.7879, *p* = 0.4490; SPC group vs. SPC + NC-shRNA group, n = 6 mice per group, two-tailed *t* test, t = 0.2775, *p* = 0.7871; Fig. 4E, F), LATS1 (IR group vs. IR + NC-shRNA group, n = 6 mice per group, two-tailed *t* test, t = 1.625, *p* = 0.1351; SPC group vs. SPC + NC-shRNA group, n = 6 mice per group, two-tailed *t* test, t = 0.4431, *p* = 0.6671; Fig. 4E, G) and YAP1(IR group vs. IR + NC-shRNA group, n = 6 mice per group, two-tailed *t* test, t = 1.119, *p* = 0.2892; SPC group vs. SPC + NC-shRNA group, n = 6 mice per group, two-tailed *t* test, t = 0.5429, *p* = 0.5991; Fig. 4E, H). Similar changes in expression of YAP1 from ischemic penumbra and hippocampal CA1 region have also been observed in the results of immunofluorescence. YAP1 expression was low in the Sham group, but increased significantly after IRI, expression level of YAP1 in the SPC group was further increased compared with that in the IR group, ^32^and YAP1 expression was significantly decreased after the treatment with the YAP1 inhibitor Verteporfin (100 mg/kg) (Fig. 4I–J). It was worth noting that increased expression of YAP1 from ischemic penumbra was accompanied by nuclear translocation of YAP1 (Fig. 4I). In addition, ischemic penumbra and hippocampus CA1 showed the least apoptosis (TUNEL^+^/Cle.caspase-3^+^) in the Sham group, increased apoptosis after IRI, decreased apoptosis after SPC, and significantly increased apoptosis after the treatment with Verteporfin (Fig. 4K–L). These results suggested that Hippo-YAP signaling pathway was activated after brain IRI, and SPC further promoted the activation, and that this activation was associated with apoptosis.

### SPC regulates mitochondrial dynamics-related proteins via Shh-YAP signaling pathway

**Fig. 5:**
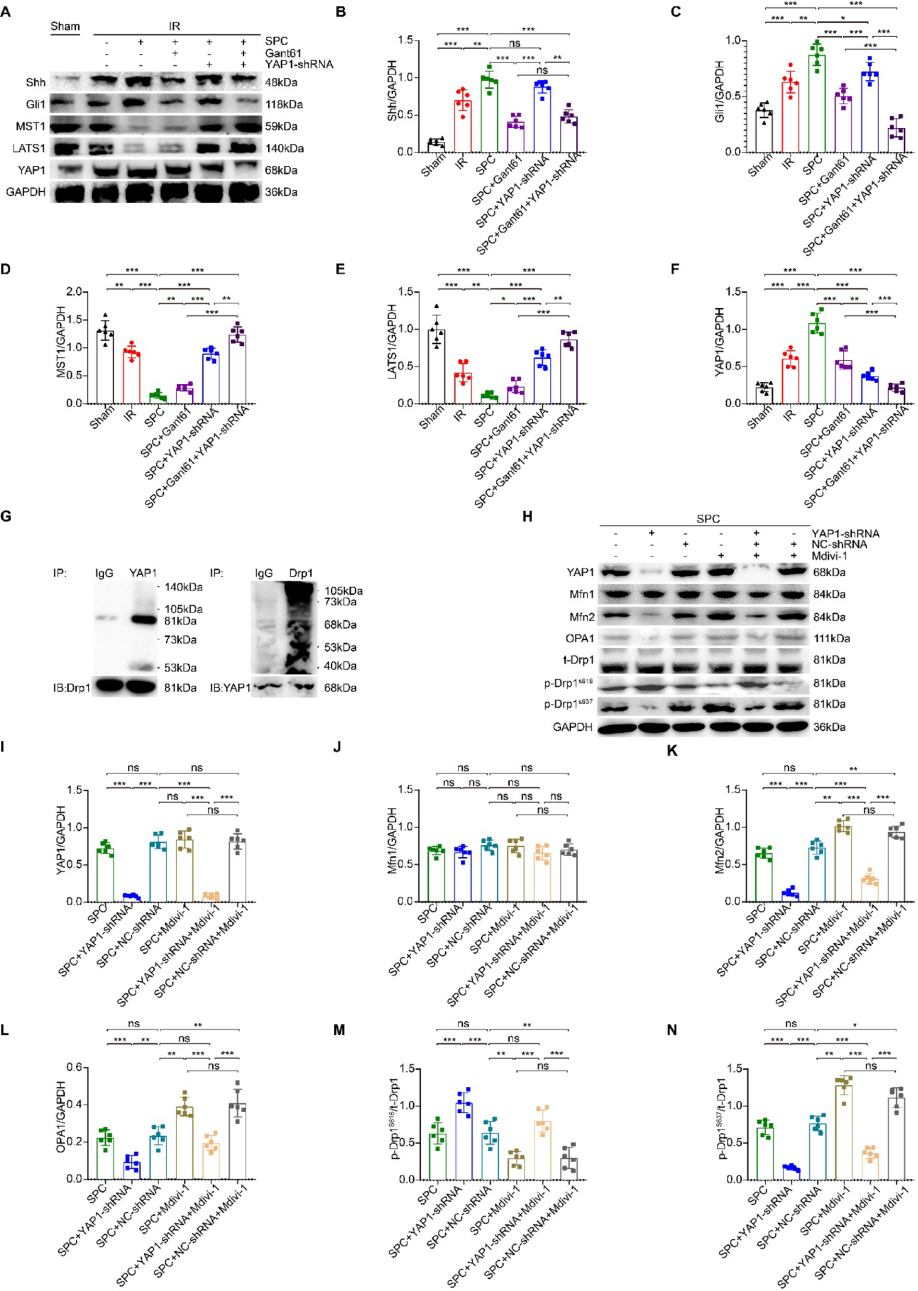
The interaction of Shh-YAP signaling pathway and mitochondrial dynamics-related proteins in hippocampus. **A**–**F** Protein expression levels and analysis of Shh signaling pathway and Hippo-YAP signaling pathway in hippocampus. **G** Results of Co-IP assays detecting endogenous YAP1 and Drp1 interaction in hippocampus. **H** Protein expression levels of YAP1, mitochondrial fusion proteins, and mitochondrial fission proteins. **I**–**N** Western blot analysis of YAP1, mitochondrial fusion proteins, and mitochondrial fission proteins. NC-shRNA was used as negative control. Immunoprecipitation (IP), Immunoblotting (IB). Data are presented as the mean ± SD. Non-significant (ns), *P < 0.05, **P < 0.01, ***P < 0.001

Considering that numerous signaling pathways form a complex homeostatic regulatory network, rather than acting in isolation. We first explored the interaction between the Shh signaling pathway and Hippo-YAP signaling pathway in hippocampus. Mice were randomized to receive AAV-YAP1-shRNA-EGFP [33], Gant61 (50 mg/kg) or AAV-YAP1-shRNA-EGFP combined with the Shh signaling pathway inhibitor Gant61. We found that the protein expression levels of Shh, Gli1 and YAP1 were similar (Fig. 5A–C, F), however, the expression trends of MST1 and LATS1 were opposite to that of YAP1 (Fig. 5A, D–F). Under normal conditions, the protein expression levels of YAP1, Shh and Gli1 were low. After IRI, the protein expression levels of YAP1 (n = 6 mice per group, two-tailed *t* test, t = 7.954, *p* < 0.0001, Fig. 5A, F), Shh (n = 6 mice per group, two-tailed *t* test, t = 9.453, *p* < 0.0001, Fig. 5A, B) and Gli1 (n = 6 mice per group, two-tailed *t* test, t = 5.259, *p* = 0.0004, Fig. 5A, C) increased, and SPC further increased the protein expression levels of them (n = 6 mice per group, two-tailed *t* test, t = 6.850, *p* < 0.0001, Fig. 5A, F; n = 6 mice per group, two-tailed *t* test, t = 3.763, *p* = 0.0037, Fig. 5A, B; n = 6 mice per group, two-tailed *t* test, t = 4.347, *p* = 0.0015, Fig. 5A, C). Administration of Gant61 resulted in decreased protein expression levels of YAP1 (n = 6 mice per group, two-tailed *t* test, t = 6.682, *p* < 0.0001, Fig. 5A, F), Shh (n = 6 mice per group, two-tailed *t* test, t = 10.020, *p* < 0.0001, Fig. 5A, B) and Gli1 (n = 6 mice per group, two-tailed *t* test, t = 7.604, *p* < 0.0001, Fig. 5A, C). However, in mice with the administration of YAP1-shRNA, there was no statistical difference in the protein expression level of Shh (n = 6 mice per group, two-tailed *t* test, t = 1.636, *p* = 0.1329, Fig. 5A, B) compared to that in the SPC group, while the protein expression level of Gli1 were decreased compared to the SPC group (n = 6 mice per group, two-tailed *t* test, t = 2.913, *p* = 0.0155, Fig. 5A, C). Additionally, YAP1 and Gli1 protein expression levels in the hippocampus of mice receiving YAP1-shRNA combination with Gant61 was lower than that of mice receiving YAP1-shRNA (n = 6 mice per group, two-tailed *t* test, t = 5.270, *p* = 0.0004, Fig. 5A, F; n = 6 mice per group, two-tailed *t* test, t = 4.987, *p* = 0.0005, Fig. 5A, C) or Gant61 (n = 6 mice per group, two-tailed *t* test, t = 6.931, *p* < 0.0001, Fig. 5A, F; n = 6 mice per group, two-tailed *t* test, t = 10.652, *p* < 0.0001, Fig. 5A, C) alone. Of note, the protein expression level of Shh in mice treated with YAP1-shRNA combined with Gant61 was lower than that of mice treated with YAP1-shRNA alone after SPC (n = 6 mice per group, two-tailed *t* test, t = 7.926, *p* < 0.0001, Fig. 5A, B), but was not statistically different from that of mice treated with Gant61 alone after SPC (n = 6 mice per group, two-tailed *t* test, t = 1.474, *p* = 0.1712, Fig. 5A, B). These results indicated that Gant61 decreased the protein expression level of Shh, while YAP1-shRNA had no effect on the protein expression level of Shh, suggesting that in the signaling pathway network after SPC, Shh is upstream of YAP1.

Drp1 is a core protein of mitochondrial fission, and we confirmed the endogenous interaction between YAP1 and Drp1 in the hippocampus after SPC by co-immunoprecipitation assays (Fig. 5G). Next [34], we used Mitochondrial division inhibitor 1 (Mdivi-1) (40 mg/kg), an inhibitor of Drp1, to clarify the relationship between YAP1 and mitochondrial dynamics-related proteins in the hippocampus. We observed that NC-shRNA had no effect on the expression levels of YAP1 and mitochondrial dynamics-related proteins (n = 6 mice per group, two-tailed *t* test, *p* > 0.05, Fig. 5H–N). The protein expression levels of Mitofusin1 (Mfn1) showed no statistically significant differences among the groups (n = 6 mice per group, one-way ANOVA, F = 1.852, *p* = 0.1328, Fig. 5H, J). YAP1 was only reduced after YAP1-shRNA was administered (n = 6 mice per group, two-tailed *t* test, t = 21.001, *p* < 0.0001, Fig. 5H–I), and Mdivi-1 did not affect YAP1 protein expression level (n = 6 mice per group, two-tailed *t* test, t = 0.4053, *p* = 0.6938, Fig. 5H–I). The protein levels of Mitofusin2 (Mfn2), which regulates mitochondrial outer membrane fusion, and Optic Atrophy 1 (OPA1), which regulates mitochondrial inner membrane fusion, were significantly decreased after YAP1-shRNA administration (n = 6 mice per group, two-tailed *t* test, t = 16.130, *p* < 0.0001, Fig. 5H, K; n = 6 mice per group, two-tailed *t* test, t = 5.952, *p* = 0.0001, Fig. 5H, L) and markedly increased after Mdivi-1 administration (n = 6 mice per group, two-tailed *t* test, t = 8.826, *p* < 0.0001, Fig. 5H, K; n = 6 mice per group, two-tailed *t* test, t = 6.303, *p* < 0.0001, Fig. 5H, L). The protein expression level of p-Drp1^S616^ was significantly increased after YAP1-shRNA was administered (n = 6 mice per group, two-tailed *t* test, t = 8.826, *p* < 0.0001, Fig. 5H, M) and significantly decreased after Mdivi-1 administration (n = 6 mice per group, two-tailed *t* test, t = 4.806, *p* = 0.0007, Fig. 5H, M). The protein expression level of p-Drp1^S637^, in contrast to that of p-Drp1^S616^, was significantly decreased after YAP1-shRNA was administered (n = 6 mice per group, two-tailed *t* test, t = 13.010, *p* < 0.0001, Fig. 5H, N) and significantly increased after Mdivi-1 administration (n = 6 mice per group, two-tailed *t* test, t = 8.628, *p* < 0.0001, Fig. 5H, N). These data suggested that YAP1 can regulate both mitochondrial fusion-related proteins and mitochondrial fission-related proteins. We already know that YAP1 is positioned downstream of Shh in the signaling network after SPC. Therefore, SPC may regulate mitochondrial dynamics-related proteins through the Shh-YAP1 signaling pathway.

### SPC improves mitochondrial structure and function via Shh-YAP signaling pathway

Neuronal mitochondrial dynamics are disturbed after cerebral IRI and mitochondrial dynamics has emerged as a critical neuroprotective target in the treatment of cerebral IRI [35]. SPC may regulate mitochondrial dynamics-related proteins through the Shh-YAP signaling pathway. Therefore, our next step was to investigate the impact of SPC on mitochondrial structure and function after cerebral IRI.

**Fig. 6:**
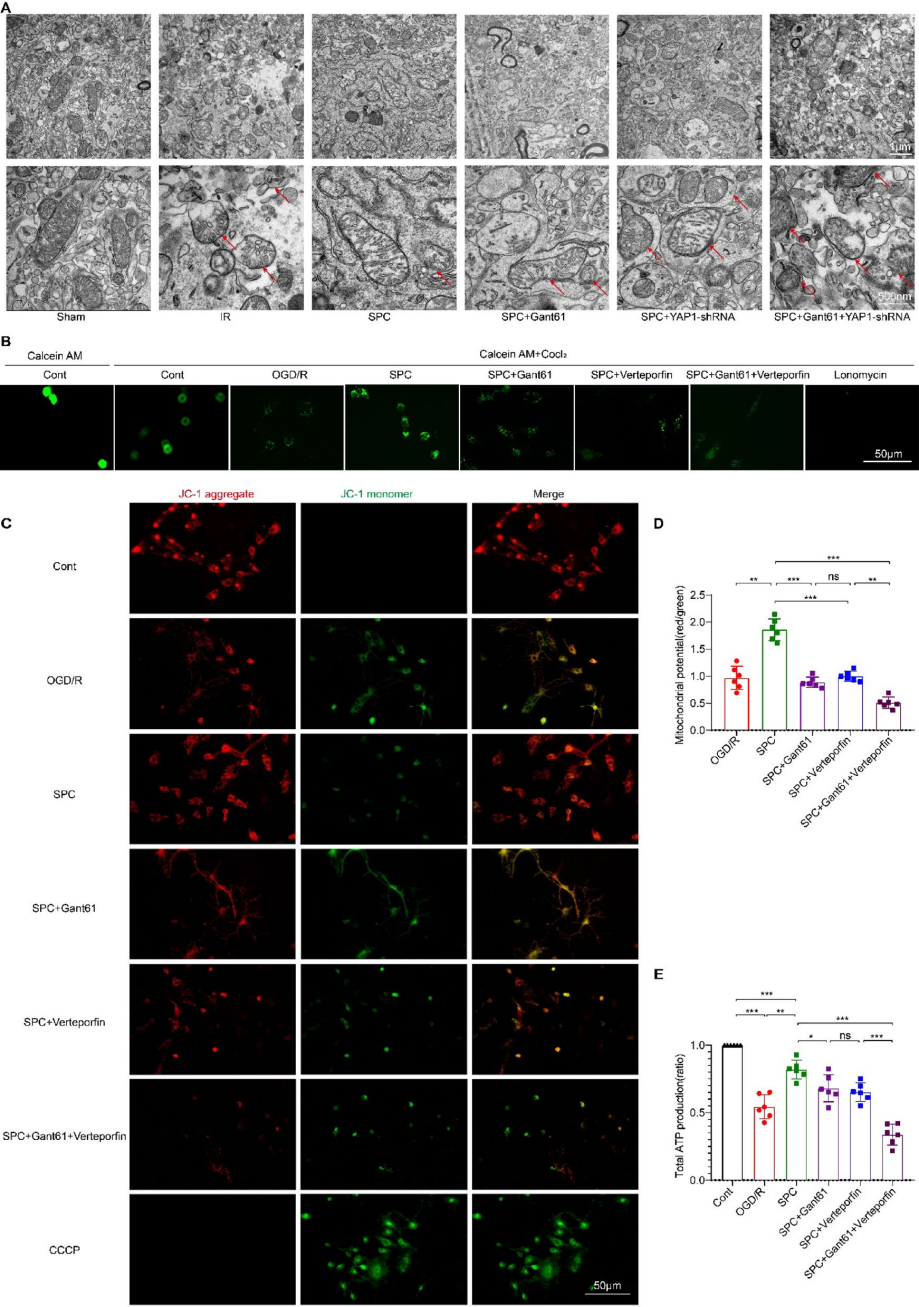
SPC improves mitochondrial structure and function via Shh-YAP signaling pathway. **A** Electron microscopy images depicting mitochondrial structure in hippocampus. Red arrowheads indicate mitochondrial swelling and disorganized mitochondrial cristae. **B** Representative images of MPTP opening using the Calcein AM and CoCl_2_ assay kit in primary cortical neurons. **C**, **D** Representative images and analysis of primary cortical neurons loaded with the mitochondrial membrane potential indicator JC-1. In healthy cells with high MMP, JC-1 forms aggregates emitting red fluorescence at 590 nm. In cells with low MMP, JC-1 remains in the monomeric form, emitting maximal green fluorescence at 527 nm. CCCP is an inhibitor of the mitochondrial electron transport chain and used as the positive control. **E** Analysis of cellular ATP production of primary cortical neurons. Data are presented as the mean ± SD. *P < 0.05, **P < 0.01, ***P < 0.001

First, we confirmed by TEM imaging that the presence of mitochondrial swelling, disorganized cristae, and fragmented, shorter mitochondria in ischemic penumbra. In contrast, there were fewer of these alterations in brain samples after SPC. Administration of Shh signaling pathway inhibitors Gant61 or YAP1-shRNA resulted in disruption of the mitochondrial structure and morphology improved by SPC. Furthermore, the damage of mitochondrial structure and morphology in the hippocampus of mice receiving YAP1-shRNA combination with Gant61 was more severe than that of mice receiving YAP1-shRNA or Gant61 alone (Fig. 6A).

Next, we conducted detection of MPTP, and changes in the opening degree of MPTP could be directly detected by Calcein AM and CoCl_2_, reflecting the situation of cell apoptosis. Calcein AM was incubated with primary cortical neurons, resulting in strong green fluorescence in the cytoplasm including mitochondria (Fig. 6B Calcein AM Cont); after further incubation with CoCl_2_, the green fluorescence of Calcein in the cytoplasm was quenched by CoCl_2_, leaving only the green fluorescence inside the mitochondria (Fig. 6B Calcein AM + CoCl_2_ Cont); treatment of cells with Ionomycin induced a large influx of extracellular Ca^2+^ into the cells, resulting in the opening of MPTP and quenching of Calcein green fluorescence, ultimately leading to cell apoptosis (Fig. 6B Calcein AM + CoCl_2_ Ionomycin). After OGD/R, the green fluorescence inside the mitochondria noticeably decreased and the opening of the MPTP was apparently augmented; SPC increased the green fluorescence and decreased the opening of the MPTP; administration of Gant61 or Verteporfin led to a reduction in the increased green fluorescence of SPC; co-administration of both inhibitors resulted in more severe damage to the green fluorescence inside the mitochondria (Fig. 6B).

We also measured MMP, which is an important parameter of mitochondrial function and has been used as an indicator of cellular health. The decrease in the red/green ratio of JC-1 reflected the reduction in MMP and impairment of mitochondrial function. Under non-stressed conditions, JC-1 formed complexes and emitted strong red fluorescence in the form of aggregates; after OGD/R, JC-1 monomers increased and emitted green fluorescence; SPC significantly reduced the green fluorescence and increased the red/green ratio of JC-1(n = 6 independent cell culture preparations per group, two-tailed *t* test, t = 7.493, *p* < 0.0001, Fig. 6C, D); administration of Gant61 (n = 6 independent cell culture preparations per group, two-tailed *t* test, t = 10.73, *p* < 0.0001, Fig. 6C, D) or Verteporfin (n = 6 independent cell culture preparations per group, two-tailed *t* test, t = 9.635, *p* < 0.0001, Fig. 6C, D) led to an increase in JC-1 monomers and a decrease in the red-green ratio of JC-1; co-administration of both inhibitors further increased the number of JC-1 monomers and decreased the red/green ratio of JC-1 (compared with SPC + Gant61, n = 6 independent cell culture preparations per group, two-tailed *t* test, t = 6.478, *p* < 0.0001; compared with SPC + Verteporfin, n = 6 independent cell culture preparations per group, two-tailed *t* test, t = 8.634, *p* < 0.0001, Fig. 6C, D).

Mitochondrial dysfunction was also identified by ATP assay. Consistently, ATP production of primary cortical neurons was significantly repressed by OGD/R injury (n = 6 independent cell culture preparations per group, two-tailed *t* test, t = 12.550, *p* < 0.0001, Fig. 6E), while it was enhanced after SPC (n = 6 independent cell culture preparations per group, two-tailed *t* test, t = 5.977, *p* = 0.0001, Fig. 6E); ATP production decreased in primary cortical neurons after Gant61 (n = 6 independent cell culture preparations per group, two-tailed *t* test, t = 2.774, *p* = 0.0197, Fig. 6E) or Verteporfin (n = 6 independent cell culture preparations per group, two-tailed *t* test, t = 4.168, *p* = 0.0019, Fig. 6E) was administered; co-administration of both inhibitors further decreased the production of ATP in primary cortical neurons (compared with SPC + Gant61, n = 6 independent cell culture preparations per group, two-tailed *t* test, t = 6.631, *p* < 0.0001; compared with SPC + Verteporfin, n = 6 independent cell culture preparations per group, two-tailed *t* test, t = 7.433, *p* < 0.0001, Fig. 6E). These results of mitochondrial structure and function detection indicated that SPC improved mitochondrial structural and functional damage through the Shh-YAP1 signaling pathway.

### SPC modulates phosphorylation and SUMOylation of Drp1 during cerebral IRI

**Fig. 7:**
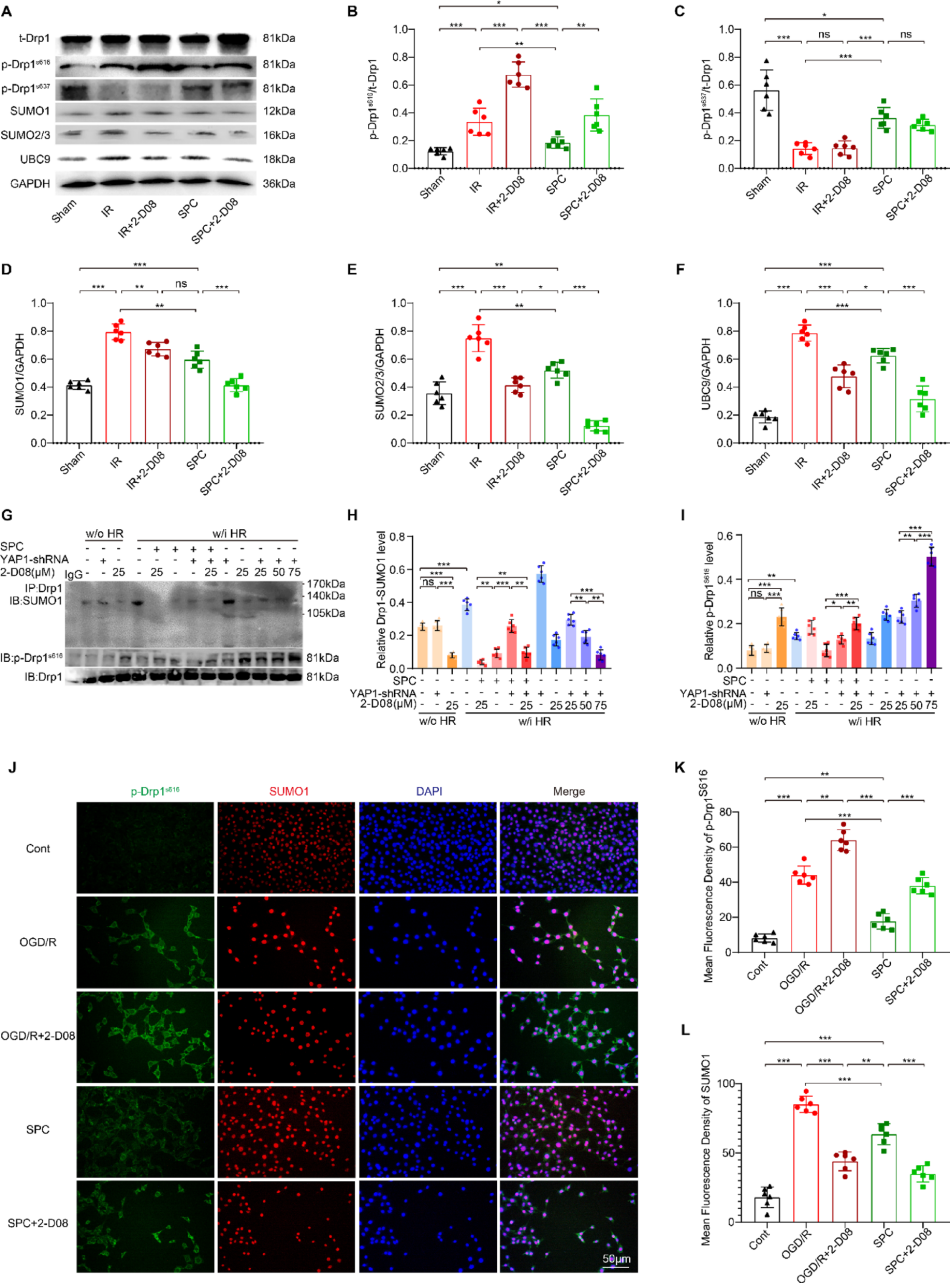
SPC modulates phosphorylation and SUMOylation of Drp1 during cerebral IRI. **A**–**F** Protein expression levels and analysis of Drp1 and SUMO-related proteins in ischemic penumbra. **G** Co-IP of SUMOylation and phosphorylation of Drp1 in HT22 cells. HT22 cells were treated with 2-D08 (24 h, 25 μM, 50 μM, 75 μM), anti-Drp1 for IP, anti-SUMO1 and anti-p-Drp1^S616^ for western blotting. **H**–**I** The quantifications of Drp1 SUMOylation and phosphorylation. **J** Immunofluorescence of p-Drp1^S616^ and SUMO1 in HT22 cells. Data are presented as the mean ± SD. Hypoxia/Reoxygenation (HR), without (w/o), with (w/i), Immunoprecipitation (IP), Immunoblotting (IB). Non-significant (ns), *p < 0.05, **p < 0.01, ***p < 0.001

To observe the effects of SPC on Drp1 and SUMOylation, we assessed the protein expression levels of phosphorylated Drp1 (p-Drp1^S616^ and p-Drp1^S637^) and SUMO-related proteins (SUMO1, SUMO2/3, UBC9) in the ischemic penumbra using Western blot. After IRI, the protein expression levels of p-Drp1^S616^ (n = 6 mice per group, two-tailed *t* test, t = 5.163, *p* = 0.0004, Fig. 7A, B), SUMO1 (n = 6 mice per group, two-tailed *t* test, t = 14.670, *p* < 0.0001, Fig. 7A, D), SUMO2/3 (n = 6 mice per group, two-tailed *t* test, t = 7.701, *p* < 0.0001, Fig. 7A, E) and UBC9 (n = 6 mice per group, two-tailed *t* test, t = 20.340, *p* < 0.0001, Fig. 7A, F) were significantly increased, while the protein expression level of p-Drp1^S637^ significantly decreased (n = 6 mice per group, two-tailed *t* test, t = 6.779, *p* < 0.0001, Fig. 7A, C). SPC decreased the protein expression level of p-Drp1^S616^ (n = 6 mice per group, two-tailed *t* test, t = 3.498, *p* = 0.0057, Fig. 7. A-B), SUMO1 (n = 6 mice per group, two-tailed *t* test, t = 5.884, *p* = 0.0002, Fig. 7A, D), SUMO2/3 (n = 6 mice per group, two-tailed *t* test, t = 5.066, *p* = 0.0005, Fig. 7A, E) and UBC9 (n = 6 mice per group, two-tailed *t* test, t = 5.131, *p* = 0.0004, Fig. 7A, F), and increased the protein expression level of p-Drp1^S637^ (n = 6 mice per group, two-tailed *t* test, t = 6.244, *p* < 0.0001, Fig. 7A, C). After IR [14], administration of the SUMOylation inhibitor 2-D08 (10 mg/kg) resulted in a significant increase in the protein expression level of p-Drp1^S616^ (n = 6 mice per group, two-tailed *t* test, t = 6.253, *p* < 0.0001, Fig. 7A, B) and a significant decrease in the protein expression level of SUMO1 (n = 6 mice per group, two-tailed *t* test, t = 4.087, *p* = 0.0022, Fig. 7A, D), SUMO2/3 (n = 6 mice per group, two-tailed *t* test, t = 7.561, *p* < 0.0001, Fig. 7A, E) and UBC9 (n = 6 mice per group, two-tailed *t* test, t = 7.606, *p* < 0.0001, Fig. 7A, F), while the protein expression level of p-Drp1^S637^ did not change significantly (n = 6 mice per group, two-tailed *t* test, t = 0.1627, *p* = 0.8740, Fig. 7A, C). These findings suggested that SPC modulates the phosphorylation of Drp1 and the expression levels of SUMO-related proteins after IRI. 2-D08 inhibited the expression levels of SUMO-related proteins, enhancing the protein expression level of p-Drp1^S616^, but it failed to affect the IRI-mediated Drp1^S637^ dephosphorylation.

Since SPC inhibited the expressions of SUMO-related proteins, the next question to explore is whether SUMOylation of Drp1 could be suppressed as well. Co-IP results of Drp1 SUMOylation and phosphorylation in HT22 cells indicated that YAP1-shRNA exerted no effect on Drp1 SUMOylation under non-IRI conditions (6 independent cell culture preparations per group, two-tailed *t* test, t = 0.8026, *p* = 0.4409, Fig. 7G, lane 3 and H), while 2-D08 decreased the protein expression level of SUMO1 (6 independent cell culture preparations per group, two-tailed *t* test, t = 8.072, *p* < 0.0001, Fig. 7G, lane 4 and H) and increased the protein expression level of p-Drp1^S616^ (6 independent cell culture preparations per group, two-tailed *t* test, t = 15.970, *p* < 0.0001, Fig. 7G, lane 4 and I). Under HR condition, the protein expression levels of Drp1 SUMOylation (6 independent cell culture preparations per group, two-tailed *t* test, t = 7.816, *p* < 0.0001, Fig. 7G, lane 5 and H) and p-Drp1^S616^ in HT22 cells increased (6 independent cell culture preparations per group, two-tailed *t* test, t = 5.777, *p* = 0.0002, Fig. 7G, lane 5 and I), while 2-D08 significantly decreased the protein expression level of SUMO1 (6 independent cell culture preparations per group, two-tailed *t* test, t = 10.54, *p* < 0.0001, Fig. 7G, lane 11 and H) and increased the protein expression level of p-Drp1^S616^ (6 independent cell culture preparations per group, two-tailed *t* test, t = 8.017, *p* < 0.0001, Fig. 7G, lane 11 and I). SPC and co-administration of SPC and 2-D08 decreased the protein expression level of SUMO1, while YAP1-shRNA significantly increased the protein expression level of SUMO1 (Fig. 7G, lane 6–10, H–I). Additionally, we found that 2-D08 also reduced the protein expression level of SUMO1 and enhanced the protein expression level of p-Drp1^S616^ in a dose-dependent manner (Fig. 7G, lane 12–14, H–I). Finally, we further confirmed the regulatory effect of SPC on p-Drp1^S616^ and SUMO1 by immunofluorescence in HT22 cells, and the protein expression trends of p-Drp1^S616^ and SUMO1 in the immunofluorescence experiments were consistent with those in Western blot (Fig. 7J–L). Therefore, these data support the functional importance of SPC in managing the homeostasis of Drp1 phosphorylation and SUMOylation during cerebral IRI.

**Fig. 8:**
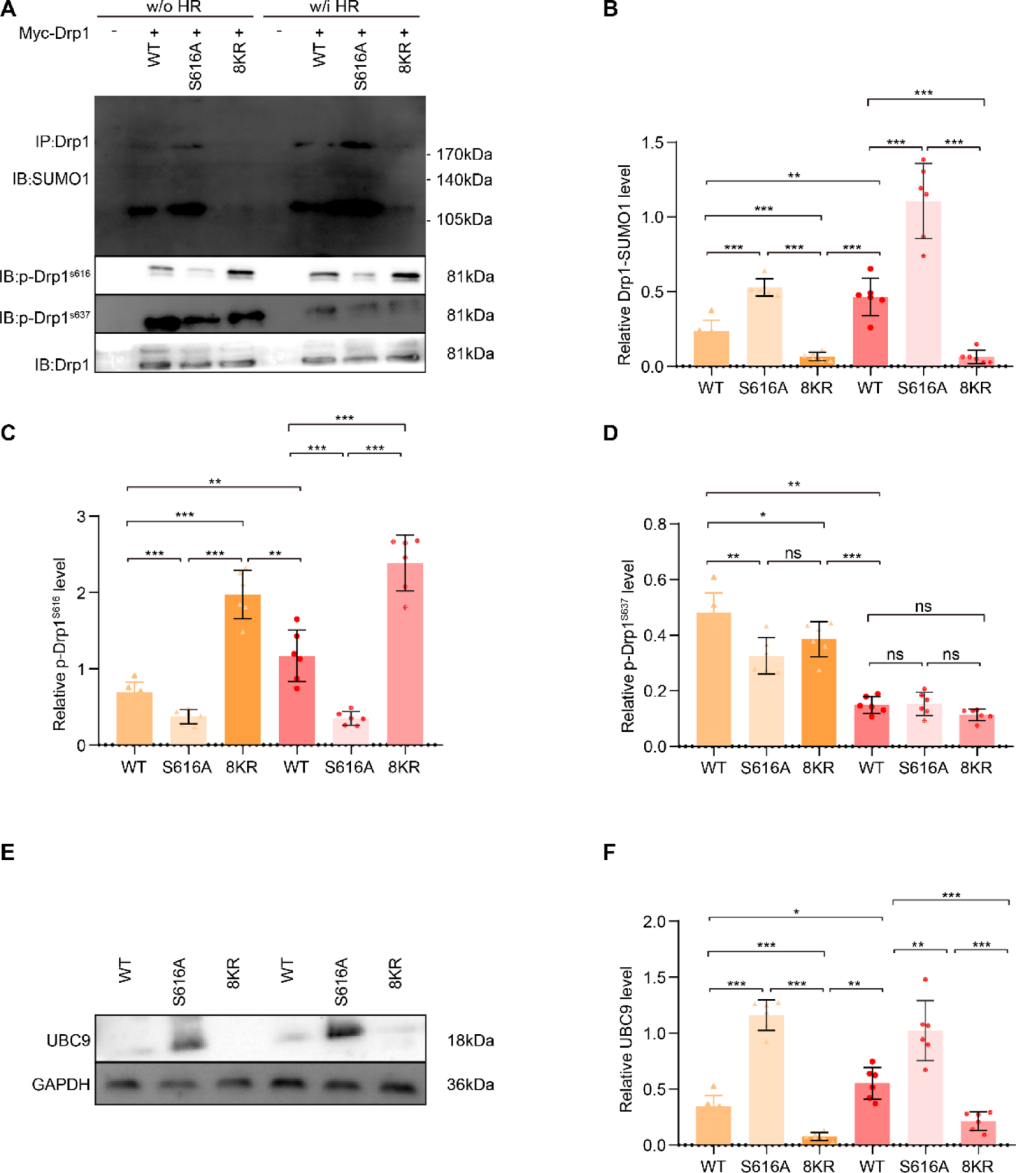
During the process of Hypoxia/Reoxygenation (HR), there is a reciprocal and compensatory relationship between the phosphorylation modification at site Ser616 of Drp1 and its SUMOylation modification. **A** SUMOylation and phosphorylation of Drp1 were analyzed by COIP in HT22 cells transfected with Myc-Drp1 or mutants of S616A and 8KR, respectively. Cells were treated w/i or w/o H/R. Anti-Drp1 immunoprecipitants were analyzed by western blotting with anti-SUMO1, anti-p-Drp1 at Ser616 and Ser637 site antibodies. **B** The quantifications of Drp1 SUMOylation, p-Drp1S616 and p-Drp1S637 in HT22 cells transfected either with Myc-Drp1 or indicated mutants. **E**–**F** Protein expression levels and analysis of UBC9 in HT22 cells. Hypoxia/Reoxygenation (HR), without (w/o), with (w/i), Immunoprecipitation (IP), Immunoblotting (IB). Non-significant (ns), *p < 0.05, **p < 0.01, ***p < 0.001

SUMOylation and phosphorylation of Drp1 were also analyzed by COIP in HT22 cells transfected with Myc-Drp1 or mutants of S616A and 8KR, respectively. Drp1 could be SUMO-modified on K532, K535, K558, K568, K594, K597, K606, K608 sites in B domain. To completely delete Drp1 SUMO1-modification, Drp1 plasmid underwent further mutation at K532R, K535R, K558R, K568R, K594R, K597R, K606R and K608R, abbreviated as Drp1-8KR (8KR). The results showed that Drp1 SUMOylation after transfecting Drp1-S616A mutant was significantly increased compared to Drp1-WT regardless of whether without Hypoxia/Reoxygenation (HR) (6 independent cell culture preparations per group, two-tailed *t* test, t = 7.623, *p* < 0.0001, Fig. 8A, B) or with HR (6 independent cell culture preparations per group, two-tailed *t* test, t = 5.594, *p* = 0.0002, Fig. 8A, B). After HR, the transfection of cells with non-SUMOylated Drp1-8KR mutant notably increased Drp1 phosphorylation at site Ser616 (6 independent cell culture preparations per group, two-tailed *t* test, t = 5.983, *p* = 0.0001, Fig. 8A, C), but not Ser637 (6 independent cell culture preparations per group, two-tailed *t* test, t = 2.042, *p* = 0.0684, Fig. 8A, D). Additionally, compared with the Drp1-WT group, after transfection with the Drp1-S616A mutant, the protein expression level of UBC9 (the only E2 ligase involved in the SUMOylation modification process) significantly increased after HR (6 independent cell culture preparations per group, two-tailed *t* test, t = 5.594, *p* = 0.0002, Fig. 8E, F). These results indicated that there might be an interplay between Drp1 phosphorylation at site Ser616 and SUMOylation, and dephosphorylation promotes SUMOylation by increasing the expression of UBC9.

## Discussion

Mechanical thrombectomy to restore cerebral blood flow in the treatment of ischemic stroke has been widely used clinically [36]. Since the surgical thrombectomy requires the use of inhalation anesthetics, the application convenience of inhalation anesthetics is evident in clinic. During the experiment, we observed that SPC significantly increased the cerebral blood flow and reduced the infarct volume after MCAO/R in mice. Subsequently, we employed flow cytometry, Nissl staining, calcium imaging and neurologic deficit scoring to assess and confirm the neuroprotective effects of SPC from various aspects including in vitro cells, tissues, in vivo cells and behavior. In this study, the experimental results first demonstrated that SPC can regulate the phosphorylation and SUMOylation of Drp1 after cerebral IRI, and there is an interaction between these modifications. SPC also regulates mitochondrial dynamics and mitochondrial function through the Shh-YAP1 signaling pathway, thereby reducing apoptosis and improving cerebral IRI. This study is the first to discover that SPC exerts neuroprotective effects on cerebral IRI through the Shh and Hippo-YAP signaling pathways, both in vivo and in vitro.

At present, the study of Shh signaling pathway in IRI remains to be further explored [8, 37, 38]. Many studies, including our previous research, have demonstrated that the Shh signaling pathway is reactivated in ischemic tissues and participates in ischemia-induced angiogenesis. For instance, a recent study reported that human umbilical vein endothelial cells exhibit angiogenic responses to both autocrine Shh and paracrine Shh secreted by astrocytes [39], but when hedgehog signaling pathway was inhibited, the pro-angiogenic effect was abolished in MCAO mice [40]. Additionally, the activation of Shh signaling pathway can also reduce apoptosis and oxidative stress after IRI. In this study, the protein expression levels of Shh and Gli1 increased after IRI, indicating activation of the Shh signaling pathway following ischemic stroke [37–40], consistent with previous research findings. Furthermore, SPC further elevated the protein expression levels of Shh and Gli1 and decreased the expression levels of pro-apoptotic proteins, suggesting that SPC functions in cerebral IRI involved in the Shh signaling pathway and apoptosis [41]. Notably, a contrary study on the role of the Shh signaling pathway in ischemic skeletal muscle suggested that endogenous Shh and ectopically administered N-terminal Shh act different effects in ischemic repair. One possible explanation is that bioavailability (expression profile and solubility) of the two sources of Shh are different.

As an evolutionarily conserved signaling pathway [42], the Hippo-YAP signaling pathway also plays a crucial role in the repair of ischemic stroke [43]. Similar to the Shh signaling pathway, the activation of the Hippo-YAP signaling pathway promoted angiogenesis and the pharmacological blockade of YAP abolished the pro-angiogenic effect after cerebral IRI [44]. In addition, the Hippo-YAP signaling pathway can interact with other signaling pathways, collectively modulating endothelial to mesenchymal transition to promote angiogenesis and stroke recovery. In th study, we also observed crosstalk between the Shh signaling pathway and the Hippo-YAP signaling pathway after ischemic stroke. With the administration of the Shh signaling pathway inhibitor Gant61 and AAV-YAP1-shRNA, we demonstrated the involvement of the Shh-YAP1 signaling pathway in the mechanism of SPC after cerebral IRI. Moreover, YAP1 knockdown or administration with the Hippo-YAP signaling pathway inhibitor Verteporfin aggravated apoptosis, while SPC exerted neuroprotective effects by further activating YAP1 to reduce apoptosis [45,46]. This discovery is similar to the previously reported findings on YAP1, which can promote cell proliferation, enhance cell survival, alleviate apoptosis and reduce inflammation in a highly activated state.

Therefore, our next investigation aims to elucidate how YAP1 regulates downstream signals to decrease apoptosis. Through Co-IP, we discovered the interaction between YAP1 and Drp1. By administering the Drp1 inhibitor Mdivi-1, we observed that YAP1 influenced the expression levels of mitochondrial dynamic-related proteins and YAP1-Drp1 axis was involved in the mechanism of SPC after cerebral IRI. Then, we observed changes in mitochondrial ultrastructure, MMP, MPTP opening and ATP production after administration with Gant61 and AAV-YAP1-shRNA or Verteporfin and found that SPC improved mitochondrial structure and function via the Shh-YAP signaling pathway. Our study exhibits partial similarities with previous researches [24]. For example, a study suggested that Irisin can mitigate gates rheumatoid arthritis by suppressing mitochondrial fission via inhibiting YAP-Drp1 signaling [47]. Another study indicated that Hydralazine protected the heart against acute IRI by inhibiting Drp1-mediated mitochondrial fission. Accordingly, this is the first detailed investigation to uncover the improvement of mitochondrial structure and function after cerebral IRI by SPC via the Shh-YAP signaling pathway.

Compared with isoflurane, sevoflurane has a smaller impact on the cardiovascular system. Elderly people have a higher risk of stroke and their cardiovascular functions are poorer, so sevoflurane is more suitable for treating stroke events [48]. A recent study has found that sevoflurane pretreatment significantly increases the expression of ATF5 in the cerebral cortex and the expression of its downstream target GDF15, which is a key regulator of mitochondrial homeostasis. Unfortunately, this protective mechanism is not activated in the elderly brain, indicating that aging weakens the body's ability to respond to mitochondrial stress responses. However, the promising findings from these animal studies have not yet been translated into improved clinical outcomes. The differences between clinical outcomes and clinical prognosis may stem from age-related mitochondrial dysfunction and other comorbidities in the elderly population, which reduces the effectiveness of anesthesia pretreatment. Therefore, it is necessary to develop strategies tailored for different age groups to reduce the risk of stroke during the perioperative period, including intervention measures targeting mitochondrial function in elderly patients.

Ischemic stroke can also induce various PTMs of proteins [49–52], such as phosphorylation, SUMOylation, ubiquitination, succinylation and methylation, which play important roles in the process of cerebral IRI. In the present study, we found that the protein expression levels of p-Drp1^S616^ and SUMO-related proteins (SUMO1, SUMO2/3, UBC9) were significantly increased in ischemic penumbra of mice, while SPC significantly decreased the protein expression levels of p-Drp1^S616^ and SUMO-related proteins [53,54]. These findings were consistent with other studies suggesting that reduced p-Drp1^S616^ and SUMOylation led to a significant reduction in both tissue and cellular damage. We also found that the phosphorylation and SUMOylation of Drp1 were interacted and modulated by SPC after cerebral IRI [14,55]. The interaction between phosphorylation and SUMOylation of Drp1 may be due to the potential enhancement of compensatory Drp1 SUMOylation after inhibition of Drp1 phosphorylation, promoting Drp1 translocation and subsequent mitochondrial fission, representing a coordinated yet independent manner to regulate Drp1 [56,57]. Additionally, SUMOylation collaborated with other PTMs to coordinately regulate pathological physiological processes. Considering the complex effects of SUMOylation, attempting to utilize SUMOylation as a therapeutic target requires comprehensive consideration of the interaction between SUMOylation and other modifications in influencing disease development.

## Conclusion

In conclusion, sevoflurane was shown to have protective effects on cerebral IRI in experimental models of ischemic stroke. Our study elucidated the molecular mechanism of the Shh signaling pathway and Hippo-YAP signaling pathway modulating mitochondrial dynamics and attenuating apoptosis in sevoflurane-mediated improvement after cerebral IRI, providing a new theoretical foundation for exploring the interactions among signaling pathways and developing novel pharmacological restorative therapeutics for cerebral IRI. Shh-YAP1 signaling pathway can be a very promising target for managing ischemic stroke.

## Supplementary Information

Below is the link to the electronic supplementary material.

Supplementary Material 1

Supplementary Material 2

## Data Availability

The research protocols and analysis plans are available in the current manuscript. All study data will be available at time of publication to researchers who provide a methodologically sound and ethically approved proposal, for any purpose of analysis.

## Abbreviations

IRI: Ischemia–reperfusion injury
SPC: Sevoflurane post-conditioning
Drp1: Dynamin-related protein 1
Shh: Sonic hedgehog
DALY: Disability adjusted life year
PTMs: Post-translational modifications
MCAO/R: Middle cerebral artery occlusion/reperfusion
OGD/R: Oxygen–glucose deprivation/reoxygenation
RRID: Research Resource Identifier (see scicrunch.org)
FBS: Fetal bovine serum
PBS: Phosphate-buffered saline
AAV: Adeno-associated viruses
GO: Gene ontology
KEGG: Kyoto encyclopedia of genes and genomes
DEGs: Differentially expressed genes
LSCI: Laser speckle contrast imaging
CBF: Cerebral blood flow
mNSS: Modified neurological severity scoring
TTC: 2,3,5-Triphenyl tetrazolium chloride
TUNEL: Terminal deoxynucleotidyl transferase-mediated dUTP nick end labeling
SDS-PAGE: Sodium dodecyl sulfate–polyacrylamide gel electrophoresis
PVDF: Polyvinylidene fluoride
Co-IP: Co-immunoprecipitation
TEM: Transmission electron microscope
ATP: Adenosine triphosphate
MMP: Mitochondrial membrane potential
MPTP: Mitochondrial permeability transition pore
PLA: Proximity ligation assay
ANOVA: Analysis of variance
Mfn2: Mitofusin2
OPA1: Optic atrophy 1
