# Supplementary material for: Sevoflurane ameliorates cerebral ischemia–reperfusion injury by modulating mitochondrial dynamics and attenuating apoptosis via Shh-YAP1 signaling pathway
Source: Mol Brain. 2026 May 21;19:57. doi: 10.1186/s13041-026-01312-3 (PMC13371202; doi:10.1186/s13041-026-01312-3)
Supplement: Supplementary file 2 — Supplementary Material 2 [file 13041_2026_1312_MOESM2_ESM.docx]

**Figure 3 H**

**The uncropped and unprocessed image of Bcl2 (26kDa) is shown as follows, and it is highlighted in red to show which part of the unmodified image has been used in the figure.**

**The uncropped and unprocessed image of Shh (48kDa) is shown as follows****, and it is highlighted in red to show which part of the unmodified image has been used in the figure.**

**The uncropped image of Gli1 (118kDa) is shown as follow****s****, and it is highlighted in red to show which part of the unmodified image has been used in the figure.**

**The uncropped image of Pro.caspase-3 (35kDa) is shown as follows, and it is highlighted in red to show which part of the unmodified image has been used in the figure.**

**The uncropped image of Cle.caspase-3 (17/19kDa) is shown as follows, and it is highlighted in red to show which part of the unmodified image has been used in the figure.**

**The uncropped image of Caspase-9 (47kDa) is shown as follows, and it is highlighted in red to show which part of the unmodified image has been used in the figure.**

**The uncropped image of Bax (21kDa) is shown as follows, and it is highlighted in red to show which part of the unmodified image has been used in the figure.**

**The uncropped image of GAPDH** **(36kDa) is shown as follows, and it is highlighted in red to show which part of the unmodified image has been used in the figure.**

**Figure 4 C**

**The uncropped image of YAP1** **(68kDa) is shown as follows, and it is highlighted in red to show which part of the unmodified image has been used in the figure.**

**The uncropped image of** **GAPDH** **(36kDa) is shown as follows, and it is highlighted in red to show which part of the unmodified image has been used in the figure.**

**Figure 4 E**

**The uncropped image of MST1 (59kDa) is shown as follows, and it is highlighted in red to show which part of the unmodified image has been used in the figure.**

**The uncropped image of** **LATS1 (140kDa) is shown as follows, and it is highlighted in red to show which part of the unmodified image has been used in the figure.**

**The uncropped image of YAP1 (68kDa) is shown as follows, and it is highlighted in red to show which part of the unmodified image has been used in the figure.**

**The uncropped image of** **GAPDH (36kDa) is shown as follows, and it is highlighted in red to show which part of the unmodified image has been used in the figure.**

**Figure 5 A**

**The uncropped image of Shh (48kDa) is shown as follows, and it is highlighted in red to show which part of the unmodified image has been used in the figure.**

**The uncropped image of** **Gli1 (118kDa) is shown as follows, and it is highlighted in red to show which part of the unmodified image has been used in the figure.**

**The uncropped image of MST1 (59kDa) is shown as follows, and it is highlighted in red to show which part of the unmodified image has been used in the figure.**

**The uncropped image of** **LATS1 (140kDa) is shown as follows, and it is highlighted in red to show which part of the unmodified image has been used in the figure.**

**The uncropped image of YAP1 (68kDa) is shown as follows, and it is highlighted in red to show which part of the unmodified image has been used in the figure.**

**The uncropped image of** **GAPDH (36kDa) is shown as follows, and it is highlighted in red to show which part of the unmodified image has been used in the figure.**

**Figure 5 G**

**IP: YAP1, IB: Drp1** **(81kDa) uncropped image.**

**IP: Drp1, IB: YAP1 (68kDa) uncropped image.**

**Figure 5 H**

**The uncropped image of YAP1 (68kDa) is shown as follows, and it is highlighted in red to show which part of the unmodified image has been used in the figure.**

**The uncropped image of Mfn1 (84kDa) is shown as follows, and it is highlighted in red to show which part of the unmodified image has been used in the figure.**

**The uncropped image of Mfn2 (84kDa) is shown as follows, and it is highlighted in red to show which part of the unmodified image has been used in the figure.**

**The uncropped image of OPA1 (111kDa) is shown as follows, and it is highlighted in red to show which part of the unmodified image has been used in the figure.**

**The uncropped image of** **t-Drp1** **(81kDa) is shown as follows, and it is highlighted in red to show which part of the unmodified image has been used in the figure.**

**The uncropped image of p-Drp1^S616^ (81kDa) is shown as follows, and it is highlighted in red to show which part of the unmodified image has been used in the figure.**

**The uncropped image of** **p-Drp1^S637^ (81kDa) is shown as follows, and it is highlighted in red to show which part of the unmodified image has been used in the figure.**

**The uncropped image of GAPDH (36kDa) is shown as follows, and it is highlighted in red to show which part of the unmodified image has been used in the figure.**

**Figure 7 A**

**The uncropped image of** **t-Drp1** **(81kDa) is shown as follows, and it is highlighted in red to show which part of the unmodified image has been used in the figure.**

**The uncropped image of p-Drp1^S616^ (81kDa) is shown as follows, and it is highlighted in red to show which part of the unmodified image has been used in the figure.**

**The uncropped image of p-Drp1^S637^ (81kDa) is shown as follows, and it is highlighted in red to show which part of the unmodified image has been used in the figure.**

**The uncropped image of** **SUMO1 (12kDa) is shown as follows, and it is highlighted in red to show which part of the unmodified image has been used in the figure.**

**The uncropped image of** **SUMO2/3 (16kDa) is shown as follows, and it is highlighted in red to show which part of the unmodified image has been used in the figure.**

**The uncropped image of** **UBC9 (18kDa) is shown as follows, and it is highlighted in red to show which part of the unmodified image has been used in the figure.**

**The uncropped image of** **GAPDH (36kDa) is shown as follows****, and it is highlighted in red to show which part of the unmodified image has been used in the figure.**

**Figure 7 G**

**IP: Drp1, IB: SUMO1 (12kDa) uncropped image uncropped image.**

**IP: Drp1, IB: p-Drp1^S616^ (81kDa) uncropped image uncropped image, and it is highlighted in red to show which part of the unmodified image has been used in the figure.**

**IB: Drp1 (81kDa) uncropped image, and it is highlighted in red to show which part of the unmodified image has been used in the figure.**

**Figure S1 A**

**IP: Drp1, IB: SUMO1 (12kDa) uncropped image uncropped image.**

**IP: Drp1, IB: p-Drp1^S616^ (81kDa) uncropped image uncropped image, and it is highlighted in red to show which part of the unmodified image has been used in the figure.**

**IP: Drp1, IB: p-Drp1^S637^ (81kDa) uncropped image uncropped image, and it is highlighted in red to show which part of the unmodified image has been used in the figure.**

**IB: Drp1 (81kDa) uncropped image, and it is highlighted in red to show which part of the unmodified image has been used in the figure.**

**Figure S1 E**

**The uncropped image of** **UBC9 (18kDa) is shown as follows, and it is highlighted in red to show which part of the unmodified image has been used in the figure.**

**The uncropped image of** **GAPDH (36kDa) is shown as follows, and it is highlighted in red to show which part of the unmodified image has been used in the figure.**
